# Emergence of a Novel Avian Pox Disease in British Tit Species

**DOI:** 10.1371/journal.pone.0040176

**Published:** 2012-11-21

**Authors:** Becki Lawson, Shelly Lachish, Katie M. Colvile, Chris Durrant, Kirsi M. Peck, Mike P. Toms, Ben C. Sheldon, Andrew A. Cunningham

**Affiliations:** 1 Institute of Zoology, Zoological Society of London, Regents Park, London, United Kingdom; 2 Edward Grey Institute, Department of Zoology, University of Oxford, South Parks Road, Oxford, United Kingdom; 3 British Trust for Ornithology, The Nunnery, Thetford, Norfolk, United Kingdom; 4 Royal Society for the Protection of Birds, The Lodge, Sandy, Bedfordshire, United Kingdom; Erasmus Medical Center, The Netherlands

## Abstract

Avian pox is a viral disease with a wide host range. In Great Britain, avian pox in birds of the Paridae family was first diagnosed in a great tit (*Parus major*) from south-east England in 2006. An increasing number of avian pox incidents in Paridae have been reported each year since, indicative of an emergent infection. Here, we utilise a database of opportunistic reports of garden bird mortality and morbidity to analyse spatial and temporal patterns of suspected avian pox throughout Great Britain, 2006–2010. Reports of affected Paridae (211 incidents) outnumbered reports in non-Paridae (91 incidents). The majority (90%) of Paridae incidents involved great tits. Paridae pox incidents were more likely to involve multiple individuals (77.3%) than were incidents in non-Paridae hosts (31.9%). Unlike the small wart-like lesions usually seen in non-Paridae with avian pox in Great Britain, lesions in Paridae were frequently large, often with an ulcerated surface and caseous core. Spatial analyses revealed strong clustering of suspected avian pox incidents involving Paridae hosts, but only weak, inconsistent clustering of incidents involving non-Paridae hosts. There was no spatial association between Paridae and non-Paridae incidents. We documented significant spatial spread of Paridae pox from an origin in south-east England; no spatial spread was evident for non-Paridae pox. For both host clades, there was an annual peak of reports in August/September. Sequencing of the avian poxvirus 4b core protein produced an identical viral sequence from each of 20 great tits tested from Great Britain. This sequence was identical to that from great tits from central Europe and Scandinavia. In contrast, sequence variation was evident amongst virus tested from 17 non-Paridae hosts of 5 species. Our findings show Paridae pox to be an emerging infectious disease in wild birds in Great Britain, apparently originating from viral incursion from central Europe or Scandinavia.

## Introduction

Avian pox is a well known disease of captive and wild birds caused by dsDNA viruses in the genus *Avipoxvirus* that affects a wide range of species globally (278 species from 70 families and 20 orders to date [1]–[3]). Avian poxvirus typically causes discrete, proliferative, ‘wart-like’ lesions on the featherless regions of the head, legs and feet. This clinical presentation is frequently self-limiting, with lesions restricted to the skin, and described as ‘dry’ pox [3]. ‘Wet’ pox, which refers to diphtheritic lesions in the alimentary or respiratory systems, has been infrequently reported in wild birds, presumably because it is more cryptic [1]. The incubation period and duration of avian poxvirus infection is variable (from a few days to many months), but affected birds with mild lesions frequently recover and this is considered to be the most common situation in wild birds [4]–[5]. Avian pox lesions, however, may compromise vision, the ability to feed, or lead to secondary bacterial or fungal infection leaving wild birds vulnerable to predation [1]. Susceptibility to avipoxvirus infection varies among host species, and in relation to host age (juveniles are most susceptible), immunocompetence, season and local environment [5].

Free-ranging great tits (*Parus major*) were first reported with florid skin lesions caused by avian pox in Norway in the early 1970s, but there are few reports of this disease in Paridae species (tits) [3]. The condition was previously described in tufted titmouse (*Baeolophus bicolor*) in the USA in the early 1960s [6], and subsequent cases have involved three great tits reported from two sites in Sweden in 2003 [7] and one report in the African blue tit (*Cyanistes teneriffae*) in the Canary Islands in 2006 [8]. Since 2005, however, there has been a marked increase in reports of avian pox in great tits, with the disease confirmed in multiple central European countries [9]. Initially, a single incident of avian pox occurred in Austria, October 2005, where four great tits in a flock of 15 birds were seen to be affected [10]. In 2007, a survey of 1819 great tits by licensed ringers in Hungary observed nodular and proliferative lesions on the head and eyelids of 15 (0.8%) birds that were confirmed or suspected to be due to avian pox [11]. Literak et al. [9] described an additional 24 cases of suspected avian pox (based on the presence of characteristic skin lesions) in great tits from December 2005 to May 2009 which comprised 19 cases (at 19 sites) in the Czech Republic, three cases (three sites) from Slovakia and two cases (two sites) from Germany.

A partial nucleotide sequence, derived from the avian poxvirus core 4b gene, was found to be identical in virus strains collected from great tits in Norway [14]–[15], Austria, Germany, Slovakia and the Czech Republic [9]–[10], [14]–[15]. These samples were found to cluster within the Canarypox virus clade, one of three distinct virus clades [12]. Adams et al. [13] found avian pox isolates from North America to form a further distinct clade. Whilst the majority of great tit avian poxvirus strains sequenced to date share 100% identity, a limited degree of genetic variation has been detected [11], [15].

In Great Britain, sporadic reports of avian pox exist from multiple wild bird families and orders [3], and the infection is considered endemic. Since the 1950s, avian pox has been reported from a number of British bird species using garden habitats, including the blackbird (*Turdus merula*), carrion crow (*Corvus corone*), chaffinch (*Fringilla coelebs*), dunnock (*Prunella modularis*), greenfinch (*Carduelis chloris*), goldfinch (*Carduelis carduelis*), house sparrow (*Passer domesticus*), jackdaw (*Corvus monedula*), starling (*Sturnus vulgaris*) and common wood-pigeon (*Columba palumbus*) [16]–[20], but no species within the Paridae family.

In this study we document the emergence and spread of this infectious disease in tit species in Great Britain. We analyse the geographic distribution, the spatial and temporal patterns of disease clustering, and the seasonality of Paridae disease incidents and compare these to patterns observed for non-Paridae pox incidents over the same period. We report on post mortem examinations performed on a subset of affected birds and the combination of histopathology, electron microscopy and PCR used for diagnosis as part of the case definition. We examine the diversity of virus isolates within British wild birds and, additionally, the phylogenetic relationship of the novel avian poxvirus of tit species in Britain to the published isolates from *P. major* in central Europe, using PCR amplification and sequencing of the avian poxvirus 4b core protein.

## Results

### Avian pox incidents

From 2006 to 2010, 302 suspected avian pox incidents were reported across Great Britain through opportunistic surveillance. Of these, 211 incidents involved Paridae species with large and severe skin lesions consistent with avian pox (two incidents in 2006, 21 in 2007, 38 in 2008, 39 in 2009 and 111 in 2010). The skin lesions were most frequently reported on the head, and less commonly on the wings, legs and other parts of the body. Descriptive terms frequently used by members of the public include “tumours”, “growths”, “swellings” and “lumps”. Where photographs were available, all skin lesions in Paridae species were consistent with avian pox. The remaining 91 incidents involved avian pox, or pox-like, lesions affecting non-Paridae species (five in 2006, 16 in 2007, 15 in 2008, 16 in 2009 and 39 in 2010). The majority of all suspected avian pox incidents (284/302, 94.0%) were reported from garden habitats with feeding stations. A minority of incidents (18/302, 6.0%) were reported from nature reserves or woodland areas, or were reports of affected birds caught by licensed ringers.

Overall, great tits were reported to be affected in 89.6% (189/211) of Paridae incidents, blue tits (*Cyanistes caeruleus*) in 18.5% (39/211) of incidents, coal tits (*Periparus ater*) in 5.2% (11/211) of incidents and marsh/willow tit (*Poecile palustris*/*Poecile montana*) in 1.4% (3/211) of incidents. Avian pox incidents in non-Paridae involved 14 species; the three most common included the dunnock (49.5%–45/91 incidents), house sparrow (13.2%–12/91 incidents) and common wood-pigeon (11.0%–10/91 incidents).

**Figure 1:**
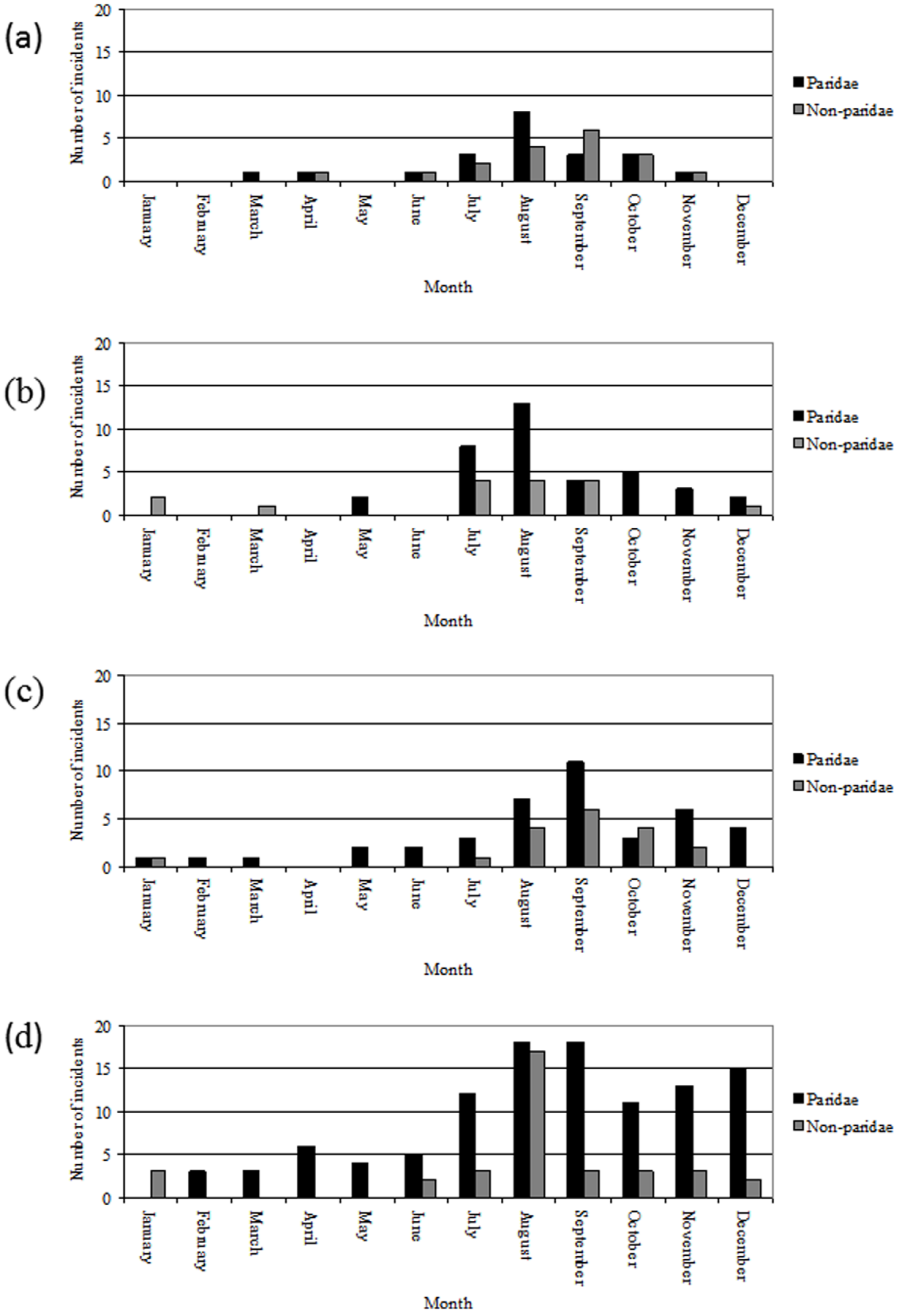
Seasonality of avian pox incidents in Paridae and non-Paridae species, 2007–2010. In 2006, a single suspect Paridae incident was reported in January and a second in August; all five non-Paridae incidents were reported from July to September inclusive.

The majority of suspected pox incidents affecting Paridae species (163/211, 77.3%) involved multiple affected individuals. This contrasts markedly with the situation for reports of avian pox in non-Paridae species over the same time period where less than a third (29/91, 31.9%) of all incidents involved multiple affected individuals. The majority of all incidents reported involved either Paridae or non-Paridae; few incidents (11/302, 3.6%) involved both Paridae and non-Paridae hosts affected together, and all of these included non-Paridae species in which avian pox has been previously reported (dunnock at four sites, blackbird at three sites, goldfinch at two sites, house sparrow at one site, and common wood-pigeon at one site). In addition, a single incident reported (with supporting photographic evidence) characteristic skin lesions of avian pox in tit species (great tit and coal tit) and a nuthatch (*Sitta europaea*), a passerine species in which avian pox has not been previously reported. Whilst incidents of avian pox in both Paridae and non-Paridae species occurred throughout the calendar year, there was a pronounced seasonal peak in reported incidents in the late summer/autumn (August–September) of each year (Figure 1).

### Pathological examinations

The index case of avian pox in British tit species occurred in a great tit from Sussex in September 2006, in which the disease was diagnosed on post-mortem examination. Avian pox was subsequently confirmed in a further 19 great tit carcases submitted from 16 incidents, 2006–2010 (see Table S1). Avian pox was diagnosed in 19 adult birds of both sexes (6 female, 3 male, 10 undetermined) and in a single nestling found dead in a nestbox. Affected birds were typically in normal (9 birds) to thin (11 birds) body condition. Avian pox was confirmed as the cause of the skin lesions in all of the suspected cases of avian pox in great tits that were examined in this study. No cases of suspected avian pox from blue tits, coal tits or marsh/willow tits were available for pathological examination.

**Figure 2:**
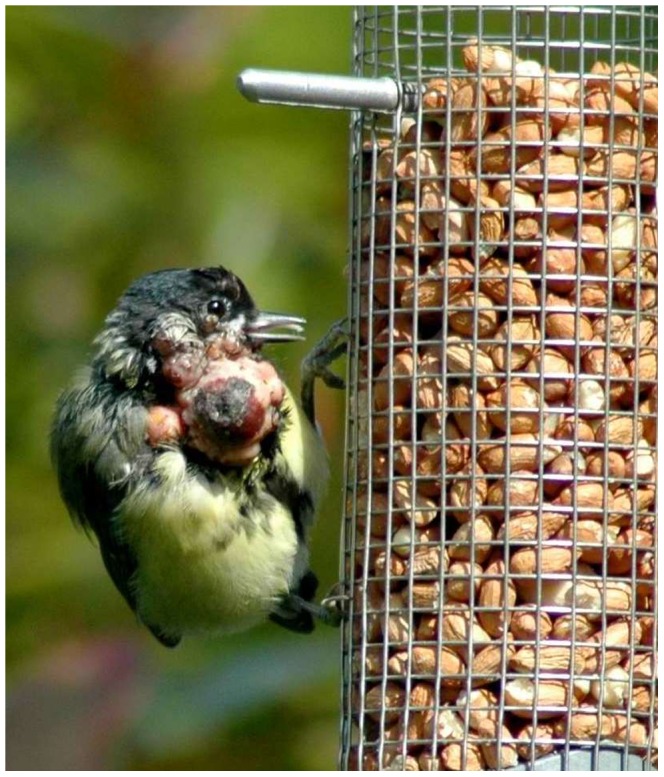
Typical avian pox lesions in a great tit at a garden feeding station (Sussex, September 2007).

Lesions were typically present on the head only (particularly affecting the scalp, periocular and perioral skin) (12 cases) and less commonly elsewhere on the head or on the body (eight cases) (Figure 2). Skin lesions were frequently large (mean dimensions 13×10×<10 mm, maximum dimensions 19×18×22 mm). The lesion surface was typically pink and featherless, sometimes with an ulcerated or scabbed surface. On cross section, the lesions had a cream or yellow-coloured caseous core with variable consistency from soft and paste-like to firm and inspissated. The majority of affected birds had either a single (7 cases), or small number (2–3) (11 cases), of skin lesions. All 20 cases presented with ‘dry’ pox, but diphtheritic oral lesions were present in a single great tit in combination with cutaneous lesions. This might have been a case of ‘wet’ pox, but the degree of autolysis precluded histopathological examination of the extra-cutaneous lesions and the affected tissue was not available for PCR to confirm the aetiology.

**Figure 3:**
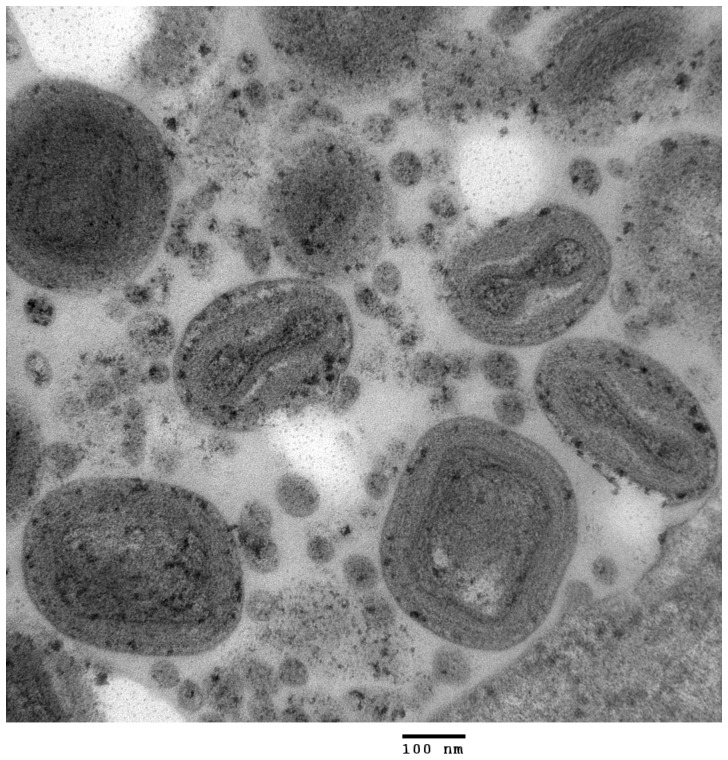
Transmission electron micrograph of avian pox virions from a skin lesion in a great tit (Case 4, Sussex, October 2007).

Histopathological examination of cutaneous lesions from 17 great tits detected lesions characteristic of avian poxvirus infection: severe hyperplasia and ballooning of epidermal cells; multiple coalescing foci of necrosis, and eosinophilic, intracytoplasmic inclusions characteristic of Bollinger bodies. The central cream- or yellow-coloured core of each lesion examined consisted of amorphous, acellular proteinaceous material (likely necrotic tissue). Electron microscopical examination of diseased tissues from two birds detected multiple intracytoplasmic virions with characteristic avipoxvirus morphology [3] (Figure 3). Poxvirus was successfully isolated from a single great tit lesion submitted for culture.

Bacteriological examination of skin lesions was performed for 13 cases. *Staphylococcus* spp. were isolated from six birds (two beta-haemolytic *Staphylococcus* sp., two *S. aureus* and two non-haemolytic *Staphylococcus* sp.). Splenomegaly, possibly in response to bacterial infection, was present in eight birds. One bird had a concurrent *Chlamydophila* sp. infection. No significant protozoal or macro-parasitic infections were identified.

Avian pox was considered to be a significant contributory factor to the cause of death for all birds examined post mortem. Injuries from predator attacks, likely the ultimate cause of death, were present in eight of the 20 birds. The periocular location of skin lesions in many cases, coupled with the large lesion size, meant that the affected eye was frequently completely obscured; affected birds would therefore have severely compromised vision and would have been vulnerable to predator attack. Large skin lesions on the wing of some birds were considered likely to have interfered with flight and therefore predator avoidance. The birds with splenomegaly may have had a bacteraemia secondary to pox which, if present, could have predisposed the birds to predation. Two birds were caught in a moribund state and were euthanased on welfare grounds due to the extent of their pox lesions.

### Patterns of spatial clustering and disease spread

**Figure 4:**
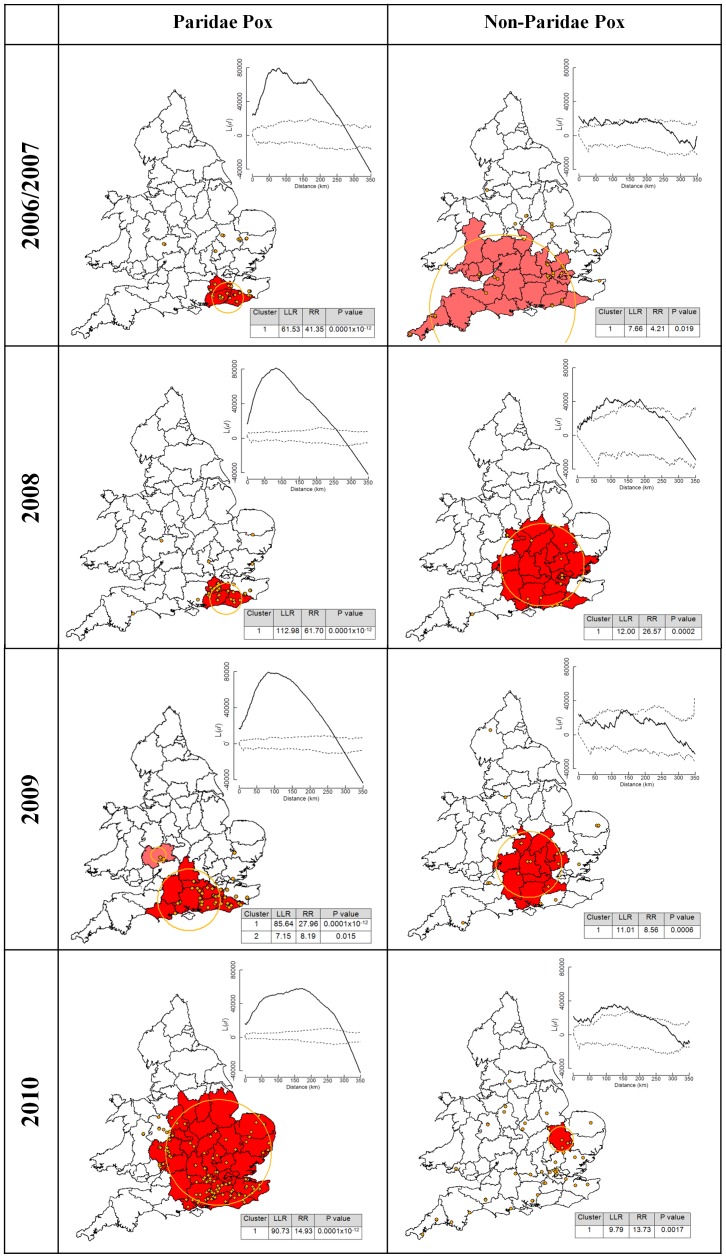
Spatial distribution and clustering patterns of avian pox in Paridae and non-Paridae hosts, 2006–2010. Maps showing the spatial distribution of avian pox in Paridae and non-Paridae hosts in each year of the study, as well as the results of K-function analysis to detect clustering of pox cases. Standardised K values (L*(d)* values, solid line) are presented as a function of increasing distance (0 to 350 km), and in relation to the 95% null simulation envelope (dotted lines). The location and spatial extent of statistically significant pox clusters accounting for heterogeneity in reporting rate (determined from SatScan spatial cluster analysis using county human household numbers as the background population) are shown by orange circles. Counties encompassed within clusters are shaded in red. Also shown are the log-likelihood ratio (LLR), the relative risk of infection (RR) and the significance (P value) of each of the identified SatScan clusters. Note that while pox cases were assigned to their county of origin for this analysis, they are plotted on these maps at their actual locations.

Results of K-function analyses provided evidence of significant spatial clustering in the distribution of suspected avian pox incidents in Paridae for each year tested, but of only weak, inconsistent clustering of avian pox incidents in non-Paridae hosts (Figure 4). K-function analysis revealed that both the geographic extent of avian pox clusters, and the distance at which clustering peaked, increased over time (in 2009, and particularly in 2010), indicative of disease spread in these years (Figure 4). No such pattern was seen for incidents of avian pox in non-Paridae hosts (Figure 4). In addition, results of bivariate K-function analysis revealed that there was no significant spatial association between the locations of Paridae pox incidents and non-Paridae pox incidents, as values of *L_ij_(d)* were contained within the 95% simulation envelope at all distances (Figure S1).

The results of the local cluster analysis indicate that the observed clustering of avian pox in Paridae was not simply an artefact of heterogeneity in reporting opportunities (as indexed by heterogeneity in household numbers across the nation; Figure 4), nor driven by underlying variation in Paridae abundance (as indexed by the relative abundance of great tits in each year across the nation; Figure S2). Both analyses revealed that throughout 2006 to 2008 avian pox incidents in Paridae were clustered in south-east England (where the index Paridae case was observed), with range expansion evident in 2009 (Figure 4 & Figure S2). In 2010, rapid further westward and northern range extension of avian pox in Paridae across Britain became evident, with the cluster analyses (accounting for both heterogeneity in reporting rate and heterogeneity in abundance) detecting a single large cluster extending into Wales in the west, and as far north as Derbyshire (Figure 4 & Figure S2). The relative risk of avian pox within the primary Paridae clusters was substantial, ranging from 15 to 73 times greater than the risk of disease at sites outside clusters (Figure 4 & Figure S2).

Significant local clusters of avian pox were also identified amongst non-Paridae hosts in each year, although in 2010 the cluster comprised just a single county (Figure 4). The relative risk of disease within non-Paridae clusters, however, was only 4 to 26 times greater than the risk of disease at sites outside clusters. Also, in contrast to the pattern revealed for Paridae hosts, there appeared to be a decrease in the spatial extent of pox clustering in non-Paridae hosts over time (Figure 4). There was no consistent overlap in the geographic locations of clusters of pox in Paridae and non-Paridae hosts (Figure 4).

**Figure 5:**
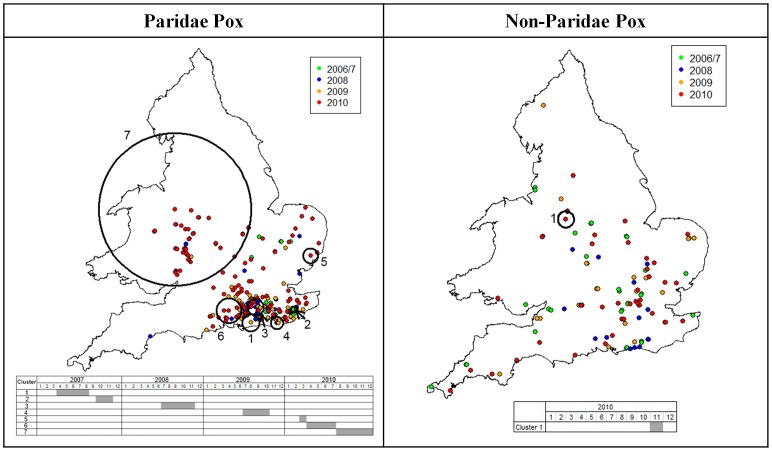
Spatio-temporal clustering of avian pox infections in Paridae and non-Paridae hosts from 2006 to 2010. Maps showing locations of avian pox incidents in Paridae and non-Paridae hosts in each year of the study, along with the locations and spatial extent of significant (P<0.05) spatiotemporal clusters detected by the space-time permutation scan statistic model. The temporal extent of clusters is given in the tables below the maps.

Results of the space-time permutation analysis confirm the above findings, showing significant spread of avian pox in Paridae in 2010, and little evidence of spread, nor any substantial space-time interaction, in avian pox amongst non-Paridae hosts (Figure 5). Initially, clusters of high avian pox incidence rates amongst Paridae were restricted to south-east England, where the index case was observed. In 2010, however, clusters of high avian pox incidence rates in Paridae shifted away from this source location, with the last cluster temporally being the most spatially distant to the index case.

As we were conservative in assigning the number of infected individuals at incidents with ‘multiple’ affected individuals (with only two cases assigned to each such site), and as incidents involving ‘multiple’ infected individuals were more frequent for Paridae hosts, the disparity between the spatial, and spatio-temporal, structuring of avian pox in these two host clades is likely to be more extreme than that revealed by our analyses.

### Molecular investigations

PCR and sequencing for avian poxvirus was performed on DNA extracts from skin lesions from 20 great tits (see Table S1) and from 17 non-Paridae British birds (see Table S2). A single identical 447 nucleotide avian poxvirus sequence was obtained from all 20 great tit samples following amplification of the 4b core protein gene (Genbank ref. JQ067665). This sequence was identical to the 4b core protein amplicon generated from great tit pox lesions in Norway (Genbank ref. AY453174 and AY453175, 1972) and central Europe, including Austria (Genbank ref. DQ857759, 2005), Hungary (Genbank ref. EF634350 and EF634349, 2006/7) and the Czech Republic (Genbank ref. FJ863096, 2007; FJ863095, 2008). This sequence corresponds to Canarypoxvirus subclade B1 as classified by Jarmin et al. [12]. As noted by Literak et al. [9], this sequence is identical to Genbank entries for avian poxvirus from unrelated bird hosts from various locations, including the Eurasian thick-knee [stone curlew] (*Burhinus oedicnemus*), the Hawaii amakihi (*Hemignathus virens*) and the apapane (*Himatione sanguinea*) (also from Hawaii) [21]–[23].

**Figure 6:**
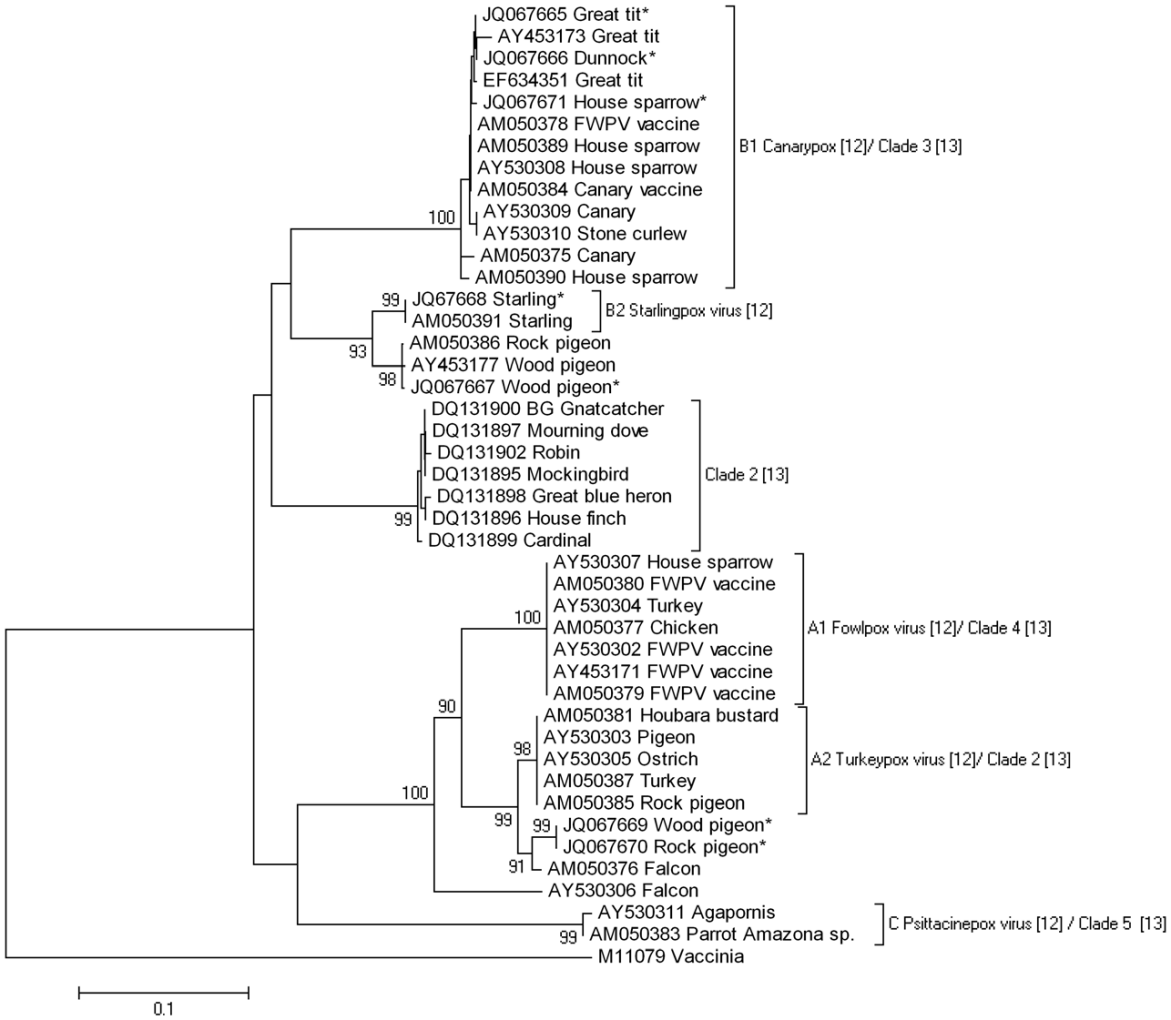
Phylogenetic analysis of avipoxvirus from British birds based on the 4b core protein. Neighbor-joining phylogram with bootstrap values (1000 replicates) of >90% shown. Sequences derived in this study are indicated with an asterisk. Details of avian pox isolates from UK non-Paridae species are in Table S2. Details of Genbank listed avian pox isolates are in Table S3. The evolutionary distances were computed using the Maximum Composite Likelihood method and are in the units of the number of base substitutions per site.

Sequence data for the 4b core protein was obtained from five non-Paridae British bird species (7 dunnocks, 2 house sparrows, 1 starling, 6 common wood-pigeons, and 1 rock pigeon (*Columbia livia*)). In the avian pox phylogeny based on a 419 nucleotide partial sequence of the 4b core protein gene, the dunnock and house sparrow clustered with the British great tit samples within the Canarypox virus subclade B1, whilst the sequences from the starling, rock pigeon and common wood-pigeons were highly divergent (Figure 6). Specifically, the seven British dunnock cases generated identical sequence data to one another and to the British great tit cases. The two house sparrow poxvirus sequences were identical and showed minimal variation from the British great tit sequence (two nucleotide substitutions; 99.5% identity). Sequences from a starling and from five common wood-pigeons in the current study and from a rock pigeon (Genbank ref. AM050386) from the UK in a previous study [12] all had 63 nucleotide substitutions and 85.0% identity to the British great tit sequence, and clustered with the Starlingpox virus subclade B2. Lesions from the remaining common wood-pigeon (ZSL Case No. 886-10; see Table S2) provided a different sequence, which was identical to that derived from the rock pigeon (ZSL Case No. 849-08); these isolates clustered most closely with a Falconpox virus isolate (Genbank ref. AM050376) from the United Arab Emirates which was classified by Jarmin et al. [12] within virus clade A3.

## Discussion

Although avian pox cases are frequently documented in a wide variety of hosts [3], pox in Paridae species had not been reported previously in Great Britain. The findings of the current study indicate that a severe form of avian pox is an emerging infectious disease of Paridae species, predominantly great tits, in Great Britain.

Unlike the usual small dry scab or wart-like lesion presentation of avian pox in non-Paridae species in Great Britain, lesions in Paridae were frequently large with a cream- or yellow-coloured caseous core. The disease was confirmed in all the great tits examined; no alternative diagnosis was reached as a cause of the proliferative skin lesions reported in tit species. Consequently, we are confident that the majority of incidents reported by the public based on lesion appearance alone were caused by avian poxvirus. Senar and Conroy [24] made a similar assumption in their investigation of avian pox in European serin (*Serinus serinus*) and classified birds with visible lesions characteristic of the disease as avian pox cases.

The results of the spatial analyses of reported incidents revealed extremely strong, localised clustering of avian pox within the Paridae, which was associated with a very high relative risk of disease. In contrast, clustering of avian pox in non-Paridae hosts was substantially weaker overall, entailed lower relative risk of disease and was, in general, much more widely distributed in space. In addition, our spatial analyses revealed there to be no systematic spatial relationship between the locations of avian pox incidents in Paridae and non-Paridae hosts. Hence, the spatial dynamics of avian pox in the two host clades are apparently independent.

Following the index case of Paridae pox in Great Britain in 2006, the disease remained clustered in south-east England until 2009, when the first indications of the spatial spread of this disease were detected. By 2010, substantial geographical spread of Paridae pox was evident, with expansion of the disease both northwards and westwards. In addition, the absolute number of reported incidents of pox in Paridae hosts increased three-fold in 2010. In the late summer/autumn of 2010 there was significant national media attention concerning infections of wild birds visiting gardens in the UK, as a result of the emergence and impact of finch trichomonosis [25]. It is possible that a portion of the apparent increase in avian pox in that year might be attributed to increased public awareness of garden bird morbidity and mortality. Indeed, the number of non-Paridae incidents also increased in 2010 (although not to the same degree). Increased public awareness and reporting of garden bird disease in 2010, however, is unlikely to explain the strong signature of geographic spread of pox in Paridae hosts in that year, especially given the difference between the number of Paridae and non-Paridae reports received. Additionally, the national nature of the media reporting and its emphasis on a different disease (finch trichomonosis) would suggest that a geographic bias in the increased ‘awareness’ and reporting of pox is unlikely. Whilst information on the emergence of avian pox in Paridae was available on the Garden Bird Health *initiative* website (www.ufaw.org.uk/gbhi.php), and through collaborating ornithological organisations, we are not aware of any national press or broadcast media reports of avian pox in wild birds during this period. Furthermore, the increased reporting of pox in non-Paridae hosts in 2010 was not similarly accompanied by strong spatial clustering, or a concurrent increase in spatial spread.

Whilst incidents were reported throughout the calendar year, there was a pronounced and repeated seasonal peak in August and September, which was present in both host clades. There are multiple factors that could contribute to this seasonality. Increased abundance in vector populations, particularly mosquitoes, in the warmer late summer months could facilitate increased disease transmission at this time of year. Mosquitoes are considered an important biting insect vector for avian poxvirus transmission [3] and UK mosquito populations typically peak in late summer [26]. The seasonality of avian pox incidents in other countries typically peaks following warm wet months, when mosquito population densities are high, although there is often variability between years [3]. A Spanish outbreak of avian pox in European serins, which began in June and ended in November 1996, displayed a similar peak of disease in September/October [24].

Alternatively, increased occurrence of avian pox disease in late summer/autumn could be facilitated by the influx of a large cohort of immunologically naïve first year birds, following the relatively synchronous breeding season that results both in increased host population density and an abundance of susceptible individuals in the population. The seasonality of avian pox is similar to that observed for finch trichomonosis, which is not vector-transmitted [25], [27]. Although the emergent pathogens and bird species are different, it is plausible that the transmission rates of both these pathogens are similarly influenced by the density-dependent effects of seasonal increases in susceptible host abundance through the influx of immunologically naïve juveniles.

Also, the congregation of birds at feeding stations could facilitate opportunities for direct and indirect virus transmission. Anthropogenic provisioning of wild birds in garden habitats is a common pastime that influences contact rates among conspecifics and alters species complements; both factors influence pathogen transmission and exposure rates [28]. Avian pox outbreaks have been observed affecting house finches (*Carpodacus mexicanus*) at feeding stations in the U.S.A. [29] with congregation at feeding stations hypothesised to have facilitated virus spread [1], [3]. Alternatively, provisioning might increase visibility of diseased birds to the reporting public. In Great Britain, in recent years, there has been a shift in garden bird feeding practice to year-round feeding, with continued provisioning during the summer months [28], [30]. It is interesting to note that Paridae pox incidents in Norway and central Europe occurred slightly later in the year, chiefly during the period October to April with a peak in December [9], [31]. However, it is plausible that this seasonality may simply reflect opportunities for observation of affected birds if the majority of supplementary feeding in these countries is restricted to the winter months. Unfortunately, it is not currently possible to determine the relative importance of different factors in driving seasonal variation in disease incidence (and presumably infection) rates, and it is likely that their importance might vary geographically and with local climate and environmental conditions.

Whilst both great tits and blue tits are amongst the most common of garden bird visitors in Britain, avian pox was far more frequently reported in the great tit than the blue tit, or other Paridae species. Whether this reflects differential species susceptibility within the family, or differential rates of exposure, remains undetermined. Skin lesions with an appearance characteristic of avian pox were observed in a number of other native tit species at multiple sites, including the blue tit, the coal tit, and the marsh/willow tit. Whilst none of these other tit species were available for post-mortem examination to confirm the diagnosis, photographs demonstrating the highly characteristic skin lesions were available from at least one incident for each species. To the authors' knowledge, this is the first time that avian pox has been documented in these species within Europe.

The great tit is a common garden visitor with an estimated British population of ∼5.7 million in 2006 [32]. The BTO/JNCC/RSPB Breeding Bird Survey 2008 [33] indicates that the great tit population in England increased by 40% between 1984 and 2009 which, coupled with their frequent use of feeding stations [34], may have influenced the transmission dynamics, and facilitated the emergence, of avian pox in this species. Blue tit and coal tit are also numerous in England and frequently use garden feeders [33]; their populations have increased by 8% and 22%, respectively, over the same 20-year period. The marsh tit and (in particular) willow tit are, however, of conservation concern following dramatic recent population declines [33]; their populations have declined by 87% and 43%, respectively, over the same time frame. These last two species use woodland rather than garden habitats [35] and, as a consequence, avian pox in these species may be more cryptic.

The severe skin lesions observed in British great tits are characteristic of pox infection observed in this species in Scandinavia [31] and in central Europe [9]–[11]. The avian pox lesions were likely to have caused significant impairment of vision or mobility to the tits examined post mortem and predation often was the ultimate cause of death. The great tits submitted for post mortem examination, however, might not have been representative of all birds with the disease. Indeed, great tits with severe skin lesions were frequently reported to continue to behave and feed normally. Longitudinal demographic studies and mark-recapture analyses are required to investigate the impact of the infection on individual survival and reproductive success, as was performed in the avian pox outbreak affecting European serins in Spain and which showed a >50% mortality rate of diseased birds within two weeks of infection [24]. Such information will be key to assessing the potential impacts of Paridae pox on host populations.

The viral sequence data obtained from post-mortem cases (a single clonal strain infecting all great tits, with 100% similarity to Paridae pox viruses from central Europe and Scandinavia, in contrast to more diverse infections in non-Paridae) are consistent with two hypotheses for the emergence of Paridae pox in Great Britain. Firstly, a novel strain of avian poxvirus might have been introduced to southern England in, or just prior to, 2006. Such an incursion could have occurred via an infected bird migrating from central Europe or Scandinavia to Great Britain: the Paridae pox situation in western continental Europe is unknown to the authors. Great tit populations in Britain & Ireland are highly sedentary, with most movement related to natal dispersal [36]. While populations elsewhere in northern Europe may undertake eruptive movements, a reduction in the occurrence of long-distance movements has been noted in some study populations [37]. Large numbers of great tits have been ringed in both Great Britain and continental Europe, but exchanges of birds between these locations have rarely been detected and no exchange of great tits has been recorded between central Europe (Austria, Czech Republic, Hungary, Slovakia) or Scandinavia and Great Britain [38]–[39]. Alternatively, the arrival of a new strain of avian poxvirus might have occurred via movement of infected vectors, such as mosquitoes, either through anthropogenic translocation or through wind-borne dispersal [40].

Secondly, a strain of avian poxvirus from another species of British bird, such as the dunnock, might have spilled over into a tit species in Great Britain, on one or more occasions, and subsequently established sustained transmission with the Paridae population. One of the dunnock samples was obtained in 2004 from Leicestershire, prior to the emergence of Paridae pox. The spatial and temporal patterns of avian pox incidents involving dunnocks, however, reflected those seen among all non-Paridae hosts, with no significant spatial association found between reports of avian pox in dunnock and in tits (see Figures S3 & S4). Hence, given the widespread spatial distribution of avian pox incidents involving dunnocks and the lack of spatial association between pox incidents in dunnocks and in tits,, it is unlikely that Paridae pox is due to virus spill-over from dunnocks.

The spatial epidemiological data presented in this study are supportive of our first hypothesis: viral incursion occurs in the region of Great Britain closest to continental Europe, i.e. south-east England, and is followed by the progressive outward expansion of the disease range in subsequent years. The incidents of avian pox involving dunnocks during this period were widely distributed across Great Britain and not clustered in south-east England (see Figure S3). It is plausible that the sequence derived from the 4b core protein of these canarypox virus isolates within subclade B1 does not provide sufficient resolution to differentiate between the British dunnock and great tit strains. Experimental cross infection of these two virus strains between the two host bird species to investigate differential species susceptibility and lesion appearance would help to explore this possibility. Full genome sequencing and identification of markers to permit finer-scale resolution between avian poxvirus strains should also be performed to gain a more complete understanding of the epidemiology of avian pox in European bird populations. Finally, it should also be remembered that the species in which clinical disease is observed may not represent the natural reservoir of the virus strain [12]. The transmission of Paridae pox from a sympatric species, in which lesions are mild, and thus more cryptic, remains a possibility that requires investigation.

## Materials and Methods

### Ethics statement

No live animals were used for this research, however, the project was reviewed and approved by the Zoological Society of London's Ethics Committee.

### Avian pox incidents

Surveillance of garden bird morbidity and mortality across Great Britain from 2006 to 2010 was achieved via opportunistic (*ad hoc*) reports obtained from the general public. The majority of these reports were received by the Royal Society for the Protection of Birds Wildlife Enquiries Unit; the remainder by the British Trust for Ornithology or directly to participating veterinary laboratories. Freshly-dead birds were submitted to these participating veterinary laboratories for pathological examination. Suspected avian pox “incidents” were defined as when one or more birds were observed with grossly visible skin lesions characteristic for the disease (based on lesion description and distribution on the body) in the same location; host species were grouped within Paridae and non-Paridae clades. All incident histories were reviewed by veterinarians. Incident selection was conservative; where the skin lesions described were more or equally consistent with alternative infectious or non-infectious aetiologies they were excluded from the study. In Great Britain, *Cnemidocoptes* sp. mites and fringilla papilloma virus [41] are common causes of proliferative and cornified skin lesions restricted to the tarsus and digits. These infections typically affect the chaffinch and have not been reported in Paridae species. When available, photographic documentation of skin lesions in non-submitted, clinically-affected live birds was reviewed by veterinarians to determine if lesions were characteristic of avian pox.

The seasonal distribution of suspected avian pox incidents in Paridae and non-Paridae species was determined for each full calendar year from 1^st^ January 2007 to 31st December 2010, based on the observed month of first observation of each incident. The species complement at each incident was summarised, along with a factor for the number of individuals affected (either ‘single’ or ‘multiple’, as accurate counts of infected individuals were not available for the majority of incidents).

### Pathological examinations

Post-mortem examinations were performed on wild birds of multiple species following a standardized protocol, as described by Robinson et al. [25]. Briefly, the species, age, sex, and body weight were recorded for each bird examined. Birds were classed as juveniles until the post-juvenile body moult was complete. First year birds beyond their post-juvenile moult and adult birds were not differentiated. Sex was assigned based on inspection of the gonads and/or plumage characteristics. Qualitative body condition scores (emaciated, thin, normal and fat) were assigned based on visual inspection of pectoral muscle and fat deposits. Systematic inspection of the external and internal body systems was performed and any gross abnormalities were described. Microbiological, parasitological and histopathological investigations were performed where indicated. The liver and small intestine, in addition to any lesions found, were routinely sampled aseptically and examined for the presence of pathogenic bacteria using a standard protocol [25], [42]. Where the state of carcass preservation was adequate, suspected avian pox skin lesions were fixed in neutral-buffered 10% formalin and were processed for histopathological examination using routine methods and Haematoxylin and Eosin stain.

Transmission electron microscopy was performed on a subset of suspected avian pox skin lesions that were fixed in 2.5% buffered gluteraldehyde and post-fixed in 1% osmium tetroxide (VWR, UK) at the University College Medical School, Royal Free Campus, using Philips 201 and 501 microscopes. Virus isolation via chorioallantoic membrane inoculation with two passages in SPF hens' eggs was performed on a single suspected avian pox skin lesion collected from a great tit (England, 2007) at the Animal Health and Veterinary Laboratories Agency, using the protocol published by Jarmin et al. [12].

In the case definition used for this study, avian pox was confirmed on the basis of characteristic gross and microscopic skin lesions (including the presence of pathognomonic large intracytoplasmic eosinophilic inclusions, known as “Bollinger bodies”) [43]–[44]. The presence of avipoxvirus was further confirmed using transmission electron microscopical examination of affected tissue and/or PCR amplification and sequencing of the core 4b protein gene from skin lesions (see details below).

### Statistical analyses of disease clustering

Spatial analyses were conducted on a dataset consisting of all avian pox-consistent incidents in Paridae and non-Paridae received opportunistically from the general public from 1^st^ January 2006 to 31st December 2010. As accurate counts of the number of infected individuals involved in each incident were not available, we adopted a conservative approach and accounted for incidents with ‘multiple’ infected individuals by assigning two cases to that location, with just a single case assigned to incident locations with a ‘single’ infected individual.

The existence and extent of spatial autocorrelation (disease clustering or second-order spatial effect) in the locations of Paridae and non-Paridae pox cases was evaluated using Ripley's K-function analysis [45]–[47]. The K-function can be defined as: 
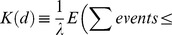
 distance *d* of an arbitrary event) where *E*( ) denotes expectation and λ is the intensity, or mean number of events per unit area. Under the assumption of complete spatial randomness arising from a homogeneous Poisson process, the expected number of events within distance *d* of an event is: 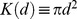 For each host clade, in each year of the study (data from 2006 and 2007 were pooled as only two cases were recorded in 2006), we evaluated the *K-*function of pox cases over a 350 km distance in 1 km increments. To facilitate interpretation of results we used a standardized version of the K function: 
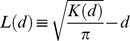
 Departures of *L(d)* from zero indicate spatial structuring (clustering if *L(d)*>0; dispersion if *L(d)*<0) and were evaluated for significance using simulated 95% confidence envelopes constructed using 999 Monte Carlo simulations of complete spatial randomness.

The bivariate K-function is an extension of the Ripley K-function that estimates the spatial dependence between two point patterns located in a defined area, by measuring the expected number of events *i* within a distance *d* of an arbitrary event *j*, divided by the overall density of the events: 
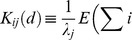
 events within distance *d* of a random *j* event).

We applied the bivariate K-function to assess spatial dependence between the locations of all Paridae and non-Paridae cases, using a standardized version of the bivariate K-function (*L_ij_(d)*, as described above). Significance of departures of *L_ij_(d)* from zero (which indicates spatial independence) were assessed using simulated 95% confidence envelopes (999 Monte Carlo simulations) constructed based on random toroidal shifts of the second point pattern (non-Paridae cases). *L_ij_(d)* values exceeding the upper 95% simulation envelope indicate aggregation or positive association between the two point patterns, while a negative association or spatial segregation is indicated by values below the lower simulation envelope. K-function analyses were performed for each host clade for each year of the study with functions in the ‘splancs’ package using R 2.12.

The Kulldorff spatial scan statistic implemented in SaTScan™ (http://satscan.org/) was used to test for the presence of significant clusters of avian pox within Britain and to identify their approximate locations and sizes [48]. The spatial scan statistic creates a series of circular windows of variable radii around every infected individual, each of which is set to contain from zero to a maximum proportion (50%) of the total population. For each location and size of the scanning window, the observed number of cases within the window is compared to the expected number of cases (given by a Poisson model and an assumption of constant risk). A likelihood ratio test is conducted to test the hypothesis that there is an elevated rate of infection within the window, when compared with the distribution outside, hence adjusting for heterogeneity in the background population. The window size and location with the maximum likelihood is defined as the most likely cluster and secondary clusters are also reported if they do not geographically overlap with another reported cluster with higher likelihood. The significance of the most likely and any secondary clusters are determined by simulated p-values using Monte Carlo methods with 999 replications, and were adjusted for the multiple testing inherent in both the many cluster locations considered, as well as the many possible collections of circles used for the scanning window [48].

To perform this spatial analysis, we assigned all pox cases to the county in which they were reported and used the number of human households per county as the background population to account for variation in reporting opportunity (such that the total population size was the total household number in GB) [49]..We are unaware of any national press or broadcast media reports of avian pox in wild birds during the study period (see Discussion), we assume that individual reporting rate is not biased by systematic spatial variation in observer awareness of pox. As above, this analysis was conducted for each host clade for each year of the study (with 2006 and 2007 data pooled). In addition, to account for the possibility that observed clustering of avian pox in Paridae was driven by underlying variation in their distribution and abundance, we also performed this analysis for the Paridae incidents only using an index of the relative abundance of great tits as the background population (such that the total population size was the sum of all the county indices). This index of Paridae abundance per county was calculated annually as the proportion of all submissions received by the British Trust for Ornithology's “Garden BirdWatch” program that included observations of great tits (the majority [∼90%] of pox incidents were observed in great tits; [50]).

Finally, the spatio–temporal interaction among cases was described using the space-time permutation scan statistic ([51]; implemented in SaTScan™), which utilises information on the spatial location and date (month of onset) of cases only. The scan statistic was defined by a cylindrical window with a circular geographic base (set to a maximum of 50% of the population, as for the purely spatial scan statistic) and with height corresponding to time (set to a maximum of six months). The number of observed cases in a cluster was compared to what would have been expected if the spatial and temporal locations of all cases were independent of each other so that there is no space-time interaction. The space-time permutation model automatically adjusts for both purely spatial and purely temporal clusters (hence the overall increase in pox cases over time is adjusted for). Significant clusters indicate geographical areas with higher infection rates than elsewhere during a given period of time.

### Molecular investigations

Frozen archived skin lesions (stored at −80°C) were collated from suspected avian pox cases in Paridae (see Table S1) and non-Paridae species (see Table S2) of British birds.. DNA was extracted from thawed skin lesions (20–25 mg) using the Biosprint 15 DNA Blood Kit (Qiagen, UK) according to the manufacturer's instructions. Molecular grade water was used as a negative control for the DNA extraction to confirm there was no contamination.

PCR was used to amplify a 578-bp product of the avipoxvirus 4b core protein gene (*fpv*167), as previously described, using the published primers 5′-CAGCAGGTGCTAAACAACAA-3′ [52] and 5′-CGGTAGCTTAACGCCGAATA-3′ [12], [14]–[15], [53]. Briefly, PCR reactions were run with 25 µL of Gotaq Colourless Mastermix (Promega, UK), 6 µL of extracted DNA (∼0.52–11.3 ug/l).), 3 µL of 10 µM forward and reverse primer and 13 µL molecular grade water to complete the 50 µL per reaction. After an initial 5 min denaturation at 94°C, 45 cycles of 94°C for 1 min, 60°C for 1 min and 72°C for 1 min were carried out, followed by a 7 min extension at 72°C using a thermal cycler (Geneamp PCR System 2500, Applied Biosystems, UK). Each PCR run contained a negative control of molecular grade water.

Amplification was confirmed visually under UV by the presence of an appropriately sized band (c. 550 bp) on an ethidium stained 2% agarose gel. PCR products were cleaned using the QIAQuick gel extraction kit (Qiagen, UK) and submitted for sequencing using the Applied Biosystems 3730 xl platform. Integrity of the DNA sequences was assessed manually by using chromatograph inspection using Chromas 2 software (www.synthesisgene.com). The sequences from both the forward primer and the reverse complement of the reverser primer PCR product were aligned for each sample using Molecular Evolutionary Genetics Analysis (MEGA) 5 software and ClustalW (www.megasoftware.net).

Sequence data were compared with available National Centre for Biotechnology Information (NCBI) Genbank entries using the Basic Local Alignment Search Tool (BLAST) search function to assess evidence for variation within the British avipoxvirus isolates and their relationship with other published avipoxviruses from Europe and the U.S.A. [9]–[13] (see Table S1).

A phylogenetic dendrogram was constructed using the Neighbour-Joining method [54] and MEGA 5 software [55]. Only common sequence across all samples (i.e. present in both forward and reverse sequence) was analysed. The percentage of replicate trees in which the associated taxa clustered together in the bootstrap test (2000 replicates) were calculated [56]. The tree was drawn to scale, with branch lengths in the same units as those of the evolutionary distances used to infer the phylogenetic tree. Evolutionary distances were computed using the Maximum Composite Likelihood method [57] in the units of the number of base substitutions per site.

## Supporting Information

Figure S1 **Bivariate K-function (** ***L_ij_*** **) plot of spatial dependence between pox infections in Paridae and non-Paridae hosts.**
(DOC)

Figure S2 **Spatial clustering patterns of avian pox in Paridae accounting for heterogeneity in population abundance.** The location and spatial extent of statistically significant pox clusters accounting for heterogeneity in population abundance (determined from SatScan spatial cluster analysis using an index of the relative abundance of great tits per county as the background population) are shown by orange circles. Counties encompassed within clusters are shaded in red. Also shown are the log-likelihood ratio (LLR), the relative risk of infection (RR) and the significance (P value) of each of the identified SatScan clusters.
(DOC)

Figure S3 **Spatial distribution and clustering patterns of avian pox in Paridae and dunnock hosts, 2006–2010.** Maps show the spatial distribution of avian pox in Paridae (orange dots) and dunnock hosts (blue dots) in each year of the study, as well as the results of K-function analysis to detect clustering of pox cases. Standardised K values (L*(d)* values, solid line) are presented as a function of increasing distance (0 to 350 km), and in relation to the 95% null simulation envelope (dotted lines). The location and spatial extent of statistically significant pox clusters accounting for heterogeneity in in reporting rate (determined from SatScan spatial cluster analysis using number of human households as the background population) are shown by orange circles. Counties encompassed within clusters are shaded in red. Also shown are the log-likelihood ratio (LLR), the relative risk of infection (RR) and the significance (P value) of each of the identified SatScan clusters. Note that while pox cases were assigned to their county of origin for this analysis, they are plotted on these maps at their actual locations.
(DOC)

Figure S4 **Bivariate K-function (** ***L_ij_*** **) plot of spatial dependence between pox infections in Paridae and dunnock hosts.** Standardized bivariate K-values (*L_ij_(d)*; solid line) are presented as a function of increasing distance, along with the 95% confidence envelope (dotted lines).
(DOC)

Table S1 **Great tit cases confirmed with avian pox (2006–2010).**
(DOC)

Table S2 **Samples of avian pox skin lesions from non-Paridae species.**
(DOC)

Table S3 **Genbank entries used in the phylogeny constructed on the 4b avian pox core protein gene.**
(DOC)

## References

[1] Hansen WR (1999) Avian pox. In: Friend M, Franson JC, editors. Field Manual of Wildlife Diseases General Field Procedures and Diseases of Birds. U.S.A.: Biological Resources Division Information and Technology Report 1999–2001, pp. 163–170.

[2] McFerran JB, McNulty MS (1993) Poxviridae. In: McFerran JB, McNulty MS, Horzinek MC, editors. Virus Infections of Birds. Oxford, UK: Elsevier Scientific Publishing, pp. 1–15.

[3] van Riper C III, Forrester DJ (2007) Avian Pox. In: Thomas NJ, Hunter DB, Atkinson CT, editors. Infectious Diseases of Wild Birds. Oxford, UK: Wiley-Blackwell Publishing, pp. 131–176.

[4] Simpson CE, Forrester DL, Nesbitt SA (1975) Avian pox in Florida Sandhill cranes. J Wildlife Dis 11: 112–115. 10.7589/0090-3558-11.1.112 163382

[5] Ritchie BW (1995) Poxviridae. In: Ritchie BW, editor. Avian viruses – Function and Control. Lake Worth, U.S.A.: Wingers Publishing Inc., pp. 285–311.

[6] Goodpasture EW, Anderson K (1962) Isolation of a wild avian pox virus inducing both cytoplasmic and nuclear inclusions. Am J Path 40: 437–454. 13900333 PMC1949548

[7] Literak I, Hromadko M, Blazkova P, Holmberg T (2004) Fagelkoppor vid Ansjon och I Narke (Avian pox in Ansjon and Orebro, Sweden, in Swedish with a summary in English) Arsrapport Annsjons, fagelstation 2003 (Lake Annsjon Bird Observatory, Sweden, Annual report 2003). Faglar I Jamtland-Hajedalen 1: 4–7.

[8] Illera JC, Emerson BC, Richardson DS (2008) Genetic characterization, distribution and prevalence of avian pox and avian malaria in the Berthelot's pipit (*Anthus berthelottis*) in Macaronesia. Parasitol Res 103: 1435–1443. 18762985 10.1007/s00436-008-1153-7

[9] Literak I, Kulich P, Robesova B, Adamik P, Roubalova E (2010) Avipoxvirus in great tits (*Parus major*). European J Wildlife Res 6: 529–534.

[10] Gruber A, Grabensteiner E, Kolodziejek J, Nowotny N, Loupal G (2007) Poxvirus in a great tit (*Parus major*). Avian Dis 51: 623–625. 17626497 10.1637/0005-2086(2007)51[623:PIIAGT]2.0.CO;2

[11] Palade EA, Biró N, Dobos-Kovács M, Demeter Z, Mándoki M, et al (2008) Poxvirus infection in Hungarian great tits (*Parus major*). Acta Veterinaria Hungarica 56: 539–546. 19149108 10.1556/AVet.56.2008.4.11

[12] Jarmin S, Manvell R, Gough RE, Laidlaw SM, Skinner MA (2006) Avipoxvirus phylogenetics: identification of a PCR length polymorphism that discriminates between the two major clades. J Gen Virol 87: 2191–2201. 16847115 10.1099/vir.0.81738-0

[13] Adams CJ, Feldman SH, Sleeman JM (2005) Phylogenetic analysis of avian poxviruses among free-ranging birds of Virginia. Avian Dis 49: 601–605. 16405007 10.1637/7369-041805R.1

[14] Weli SC, Okeke MI, Tryland M, Nilssen O, Traavik T (2004a) Characterization of avipoxviruses from wild birds in Norway. Can J Vet Res 68: 140–145. 15188959 PMC1142158

[15] Weli SC, Traavik T, Tryland M, Coucheron DH, Nilssen O (2004b) Analysis and comparison of the 4b core protein gene of avipoxviruses from wild birds: evidence for interspecies spatial phylogenetic variation. Arch Virol 149: 2035–2046. 15290371 10.1007/s00705-004-0357-0

[16] Jennings AR (1954) Diseases in wild birds. J Comp Path 64: 356–359. 13211863 10.1016/s0368-1742(54)80036-1

[17] Edwards GR (1955) Excrescenses about the eyes and on the legs and feet of dunnocks. British Birds 48: 186–187.

[18] Poulding RH (1960) Fowlpox in a carrion crow. British Birds 53: 174–175.

[19] Blackmore DK, Keymer IF (1969) Cutaneous diseases of wild birds in Britain. British Birds 62: 316–331.

[20] Pennycott TW (2003) Scaly leg, papillomas and pox in wild birds. Vet Rec 152: 444. 12708601

[21] Lüschow D, Hoffman T, Hafez HM (2004) Differentiation of avian poxvirus strains on the basis of nucleotide sequences of 4b gene fragment. Avian Dis 48: 453–462. 15529967 10.1637/7111

[22] Lierz M, Bergmann V, Czerny CP, Luschow D, Mwanzia J, et al (2007) Recurrence of avipox-infection in a collection of captive stone curlew (*Burhinus oedincnemus*). J Avian Med Surg 21: 50–55. 18069171 10.1647/1082-6742(2007)21[50:AIIACO]2.0.CO;2

[23] Jarvi SI, Triglia D, Giannoulis A, Farias M, Bianchi K, et al (2008) Diversity, origins and virulence of avipoxvirus in Hawaiian forest birds. Conserv Genet 9: 339–348.

[24] Senar JC, Conroy MJ (2004) Multi-state analysis of the impacts of avian pox on population of Serins (*Serinus serinus*): the importance of estimating recapture rates. Animal Biodiversity and Conservation 27: 133–146.

[25] Robinson RA, Lawson B, Toms MP, Peck KM, Kirkwood JK, et al (2010) Emerging infectious disease leads to rapid population declines of common British birds. PLoS ONE 5 (8) e12215 doi:10.1371/journal.pone.0012215. 20805869 10.1371/journal.pone.0012215 PMC2923595

[26] Snow KR (1990) Mosquitoes. Naturalist's Handbooks Series. Richmond Publishers, London. pp. 1–68.

[27] Lawson B, Robinson RA, Colvile K, Peck KM, Chantrey J, et al (2012) Pennycott, The emergence and spread of finch trichomonosis in the British Isles. Phil Trans Roy Soc B 367: 2852–2863. 22966140 10.1098/rstb.2012.0130 PMC3427565

[28] Robb GM, McDonald RA, Chamberlain DE, Bearhop S (2008) Food for thought: supplementary feeding as a driver of ecological change in avian populations. Front Ecol Environ 6: 476–484.

[29] McClure HE (1989) Epizootic lesions of house finches in Ventura County, California. J Field Ornithol 60: 421–430.

[30] Davies ZG, Fuller RA, Loram A, Irvine KN, Sims V, et al (2009) A national scale inventory of resource provision for biodiversity within domestic gardens. Biol Cons 142: 761–771.

[31] Holt G, Krogsrud J (1973) Pox in wild birds. Acta Veterinaria Scandinavica 14: 201–203. 4354109 10.1186/BF03547423 PMC8559852

[32] Newson SE, Evans KL, Noble DG, Greenwood JJD, Gaston KJ (2008) Use of distance sampling to improve estimates of national population sizes for common and widespread breeding birds in the UK. J Appl Ecol 45: 1330–1338.

[33] Baillie SR, Marchant JH, Leech DI, Renwick AR, Eglington SM, et al. (2012) BirdTrends 2011. BTO Research Report No. 609. BTO, Thetford. http://www.bto.org/birdtrends. Accessed 2012 Jan 4.

[34] Chamberlain DE, Vickery JA, Glue DE, Robinson RA, Conway GJ, et al (2005) Annual and seasonal trends in the use of garden feeders by birds in winter. Ibis 147: 563–575.

[35] Perrins C (1979) British Tits. The New Naturalist, Collins, London, pp.1–297.

[36] Wernham C, Toms M, Marchant J, Clark J, Siriwardena G, et al (2002) The Migration Atlas: Movements of the birds of Britain & Ireland. T & A D Poyser

[37] van Balen JH, Hage F (1989) The effect of environmental factors on tit movements. Ornis Scandinavica 20: 99–104.

[38] Robinson RA, Clark JA (2011) The Online Ringing Report: Bird ringing in Britain & Ireland in 2010 BTO, Thetford (http://www.bto.org/ringing-report, created on 22-July-2011).

[39] Gosler A (2002) The Migration Atlas: Movements of the Birds of Britain and Ireland. Thetford, UK: British Trust for Ornithology, pp. 1–900.

[40] Service MW (1997) Mosquito (Diptera: Culicidae) dispersal-the long and short of it. J Med Entomol 34: 579–588. 9439109 10.1093/jmedent/34.6.579

[41] Pennycott TW (2003) Scaly leg, papillomas and pox in wild birds. Vet Rec 152: 444. 12708601

[42] Lawson B, Howard T, Kirkwood JK, Macgregor SK, Perkins M, et al (2010) The epidemiology of salmonellosis in garden birds in England and Wales, 1993 to 2003. Ecohealth 7: 294–306. 20945078 10.1007/s10393-010-0349-3

[43] Wooduff CE, Goodpasture EW (1930) The relation of the virus of fowlpox to the specific cellular inclusions of the disease. Am J Pathol 6: 713–720. 19969937 PMC2007351

[44] Kirmse P (1966) New wild bird hosts for poxviruses. Bulletin for the Wildlife Disease Association 2: 303–33.

[45] Ripley BD (1976) The second-order analysis of stationary point processes. J Appl Probab 13: 255–266.

[46] Ripley BD (1977) Modelling spatial patterns. J R Stat Soc: series B 39: 172–212.

[47] Ripley BD (1979) Tests of ‘randomness’ for spatial point patterns. J R Stat Soc: series B 41: 368–374.

[48] Kulldorff M, Nagarwalla N (1995) Spatial disease clusters: Detection and inference. Statistics in Medicine 14: 799–810. 7644860 10.1002/sim.4780140809

[49] Census 2001 (2002) 2001 Census in England and Wales. Available: http://www.statistics.gov.uk/census2001/census2001.asp. Accessed January 13 2010.

[50] Cannon AR, Chamberlain DE, Toms MP, Hatchwell BJ, Gaston KJ (2005) Trends in the use of private gardens by wild birds in Great Britain 1995–2002. J Appl Ecol 42: 659–671.

[51] Kulldorff M, Heffernan R, Hartman J, Assunção RM, Mostashari F (2005) A space-time permutation scan statistic for the early detection of disease outbreaks. PLoS Medicine 2: 216–224. 10.1371/journal.pmed.0020059 PMC548793 15719066

[52] Binns MM, Boursnell MEG, Tomley FM, Campbell J (1989) Analysis of the fowlpoxvirus gene encoding the 4b core polypeptide and demonstration that it possesses efficient promoter sequences. Virology 170: 288–291. 2541544 10.1016/0042-6822(89)90380-2

[53] Lee LW, Lee KH (1997) Application of the polymerase chain reaction for the diagnosis of fowl poxvirus infection. J Virol Methods 63: 113–119. 9015281 10.1016/s0166-0934(96)02119-2

[54] Saitou N, Nei M (1987) The neighbor-joining method: A new method for reconstructing phylogenetic trees. Mol Biol Evol 4: 406–425. 3447015 10.1093/oxfordjournals.molbev.a040454

[55] Tamura K, Peterson D, Peterson N, Stecher G, Nei M, et al (2011) MEGA5: Molecular Evolutionary Genetics Analysis using Maximum Likelihood, Evolutionary Distance, and Maximum Parsimony Methods. Mol Biol Evol 28: 2731–2739. 21546353 10.1093/molbev/msr121 PMC3203626

[56] Felsenstein J (1985) Confidence limits on phylogenies: An approach using the bootstrap. Evolution 39: 783–791. 28561359 10.1111/j.1558-5646.1985.tb00420.x

[57] Tamura K, Nei M, Kumar S (2004) Prospects for inferring very large phylogenies by using the neighbor-joining method. Proc Natl Acad Sci USA 101: 11030–11035. 15258291 10.1073/pnas.0404206101 PMC491989

