# Supplementary material for: Emergence of a Novel Avian Pox Disease in British Tit Species
Source: PLoS One. 2012 Nov 21;7(11):e40176. doi: 10.1371/journal.pone.0040176 (PMC3504035; doi:10.1371/journal.pone.0040176)
Supplement: Figure S1 — Bivariate K-function ( Lij ) plot of spatial dependence between pox infections in Paridae and non-Paridae hosts. (DOC) [file pone.0040176.s001.doc]

**Figure S1:** Bivariate K-function (*L_ij_*) plot of spatial dependence between pox infections in Paridae and non-Paridae hosts. Standardized bivariate K-values (*L_ij_(d);* solid line) are presented as a function of increasing distance, along with the 95% confidence envelope (dotted lines).

**
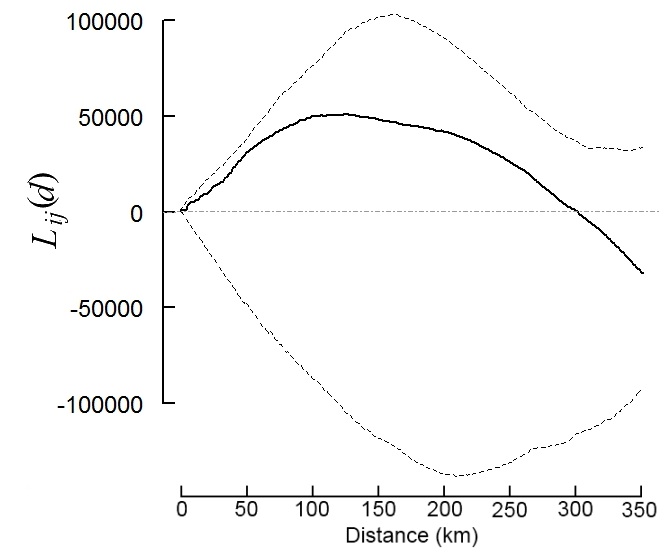
**
