# Supplementary material for: Emergence of a Novel Avian Pox Disease in British Tit Species
Source: PLoS One. 2012 Nov 21;7(11):e40176. doi: 10.1371/journal.pone.0040176 (PMC3504035; doi:10.1371/journal.pone.0040176)

**Figure S2:** Spatial distribution and clustering patterns of avian pox in Paridae accounting for heterogeneity in population abundance. The location and spatial extent of statistically significant pox clusters accounting for heterogeneity in population abundance (determined from SatScan spatial cluster analysis using an index of the relative abundance of great tits per county as the background population) are shown by orange circles. Counties encompassed within clusters are shaded in red. Also shown are the log-likelihood ratio (LLR), the relative risk of infection (RR) and the significance (P value) of each of the identified SatScan clusters.


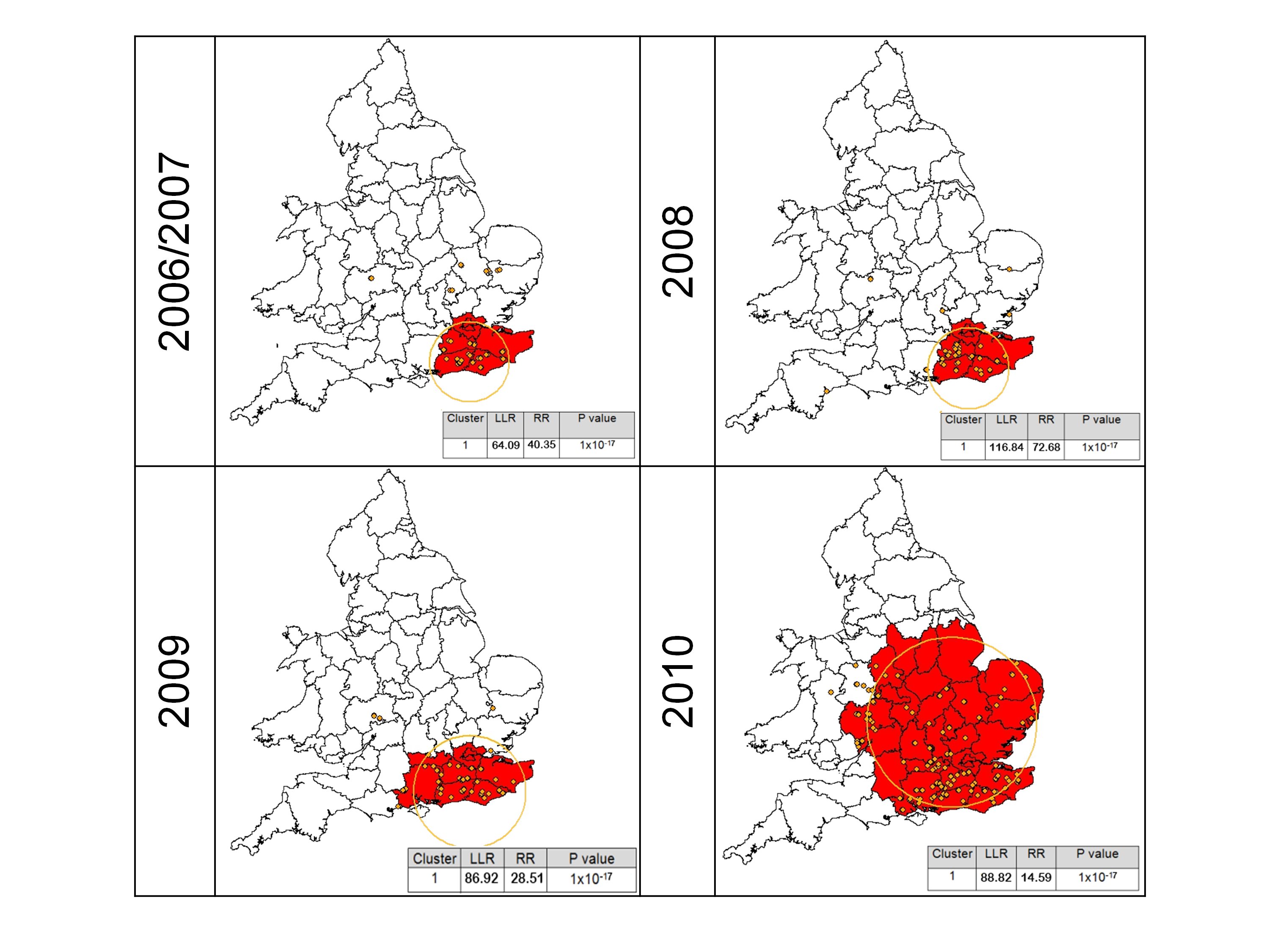

Supplement: Figure S2 — Spatial clustering patterns of avian pox in Paridae accounting for heterogeneity in population abundance. The location and spatial extent of statistically significant pox clusters accounting for heterogeneity in population abundance (determined from SatScan spatial cluster analysis using an index of the relative abundance of great tits per county as the background population) are shown by orange circles. Counties encompassed within clusters are shaded in red. Also shown are the log-likelihood ratio (LLR), the relative risk of infection (RR) and the significance (P value) of each of the identified SatScan clusters. (DOC) [file pone.0040176.s002.doc]
