# Supplementary material for: Emergence of a Novel Avian Pox Disease in British Tit Species
Source: PLoS One. 2012 Nov 21;7(11):e40176. doi: 10.1371/journal.pone.0040176 (PMC3504035; doi:10.1371/journal.pone.0040176)
Supplement: Figure S3 — Spatial distribution and clustering patterns of avian pox in Paridae and dunnock hosts, 2006–2010. Maps show the spatial distribution of avian pox in Paridae (orange dots) and dunnock hosts (blue dots) in each year of the study, as well as the results of K-function analysis to detect clustering of pox cases. Standardised K values (L(d) values, solid line) are presented as a function of increasing distance (0 to 350 km), and in relation to the 95% null simulation envelope (dotted lines). The location and spatial extent of statistically significant pox clusters accounting for heterogeneity in in reporting rate (determined from SatScan spatial cluster analysis using number of human households as the background population) are shown by orange circles. Counties encompassed within clusters are shaded in red. Also shown are the log-likelihood ratio (LLR), the relative risk of infection (RR) and the significance (P value) of each of the identified SatScan clusters. Note that while pox cases were assigned to their county of origin for this analysis, they are plotted on these maps at their actual locations. (DOC) [file pone.0040176.s003.doc]

**Figure S3:** Spatial distribution and clustering patterns of avian pox in Paridae and dunnock hosts, 2006-2010. Maps show the spatial distribution of avian pox in Paridae (orange dots) and dunnock hosts (blue dots) in each year of the study, as well as the results of K-function analysis to detect clustering of pox cases. Standardised K values (L*(d)* values, solid line) are presented as a function of increasing distance (0 to 350km), and in relation to the 95% null simulation envelope (dotted lines). The location and spatial extent of statistically significant pox clusters accounting for heterogeneity in in reporting rate (determined from SatScan spatial cluster analysis using number of human households as the background population) are shown by orange circles. Counties encompassed within clusters are shaded in red. Also shown are the log-likelihood ratio (LLR), the relative risk of infection (RR) and the significance (P value) of each of the identified SatScan clusters. Note that while pox cases were assigned to their county of origin for this analysis, they are plotted on these maps at their actual locations.

|  | **Paridae Pox** | **Dunnock Pox** |
| --- | --- | --- |
| **2006/2007** | **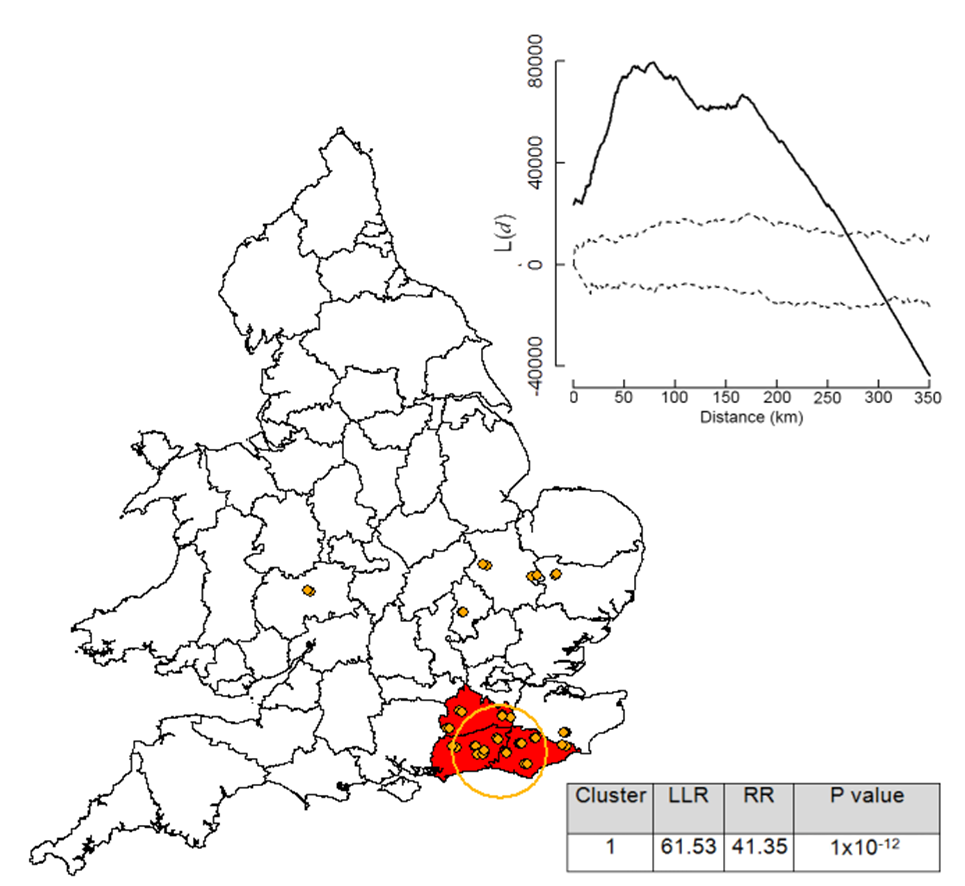** | **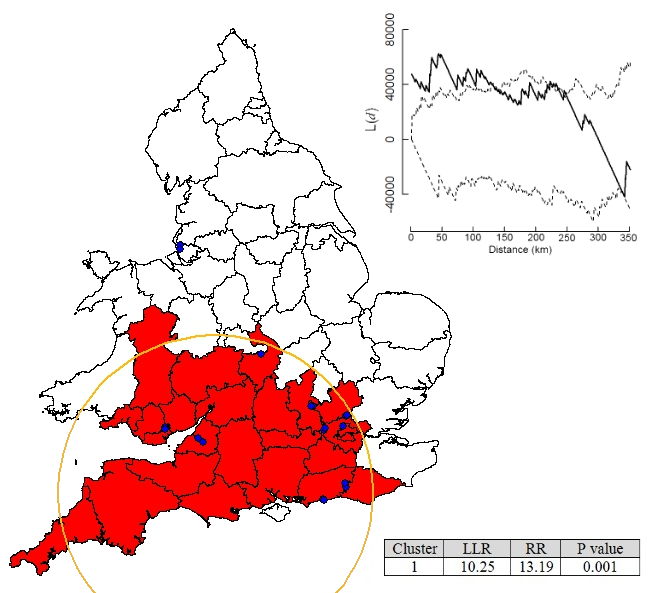** |
| **2008** | **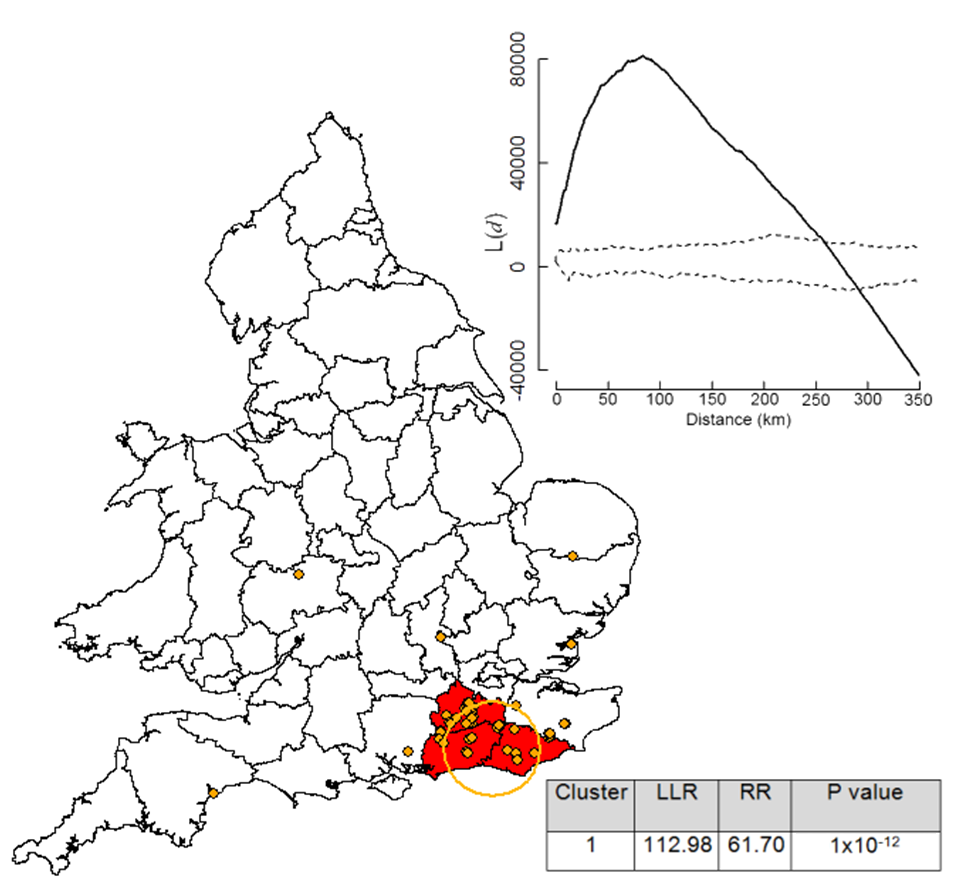** | **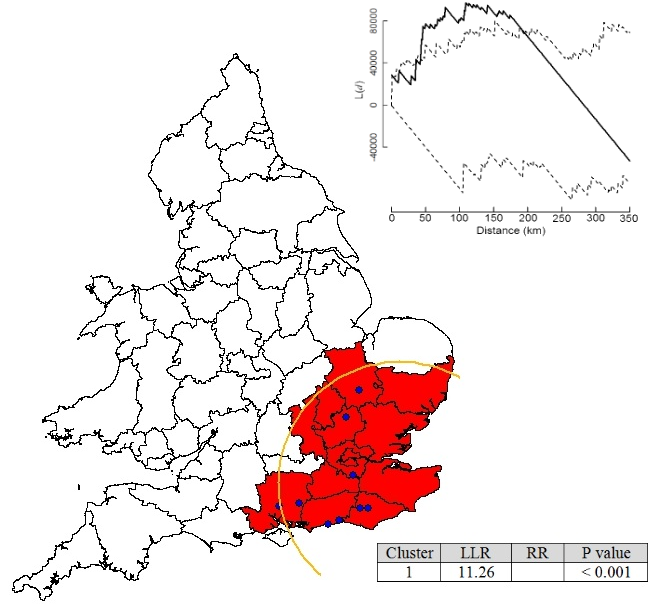** |
| **2009** | **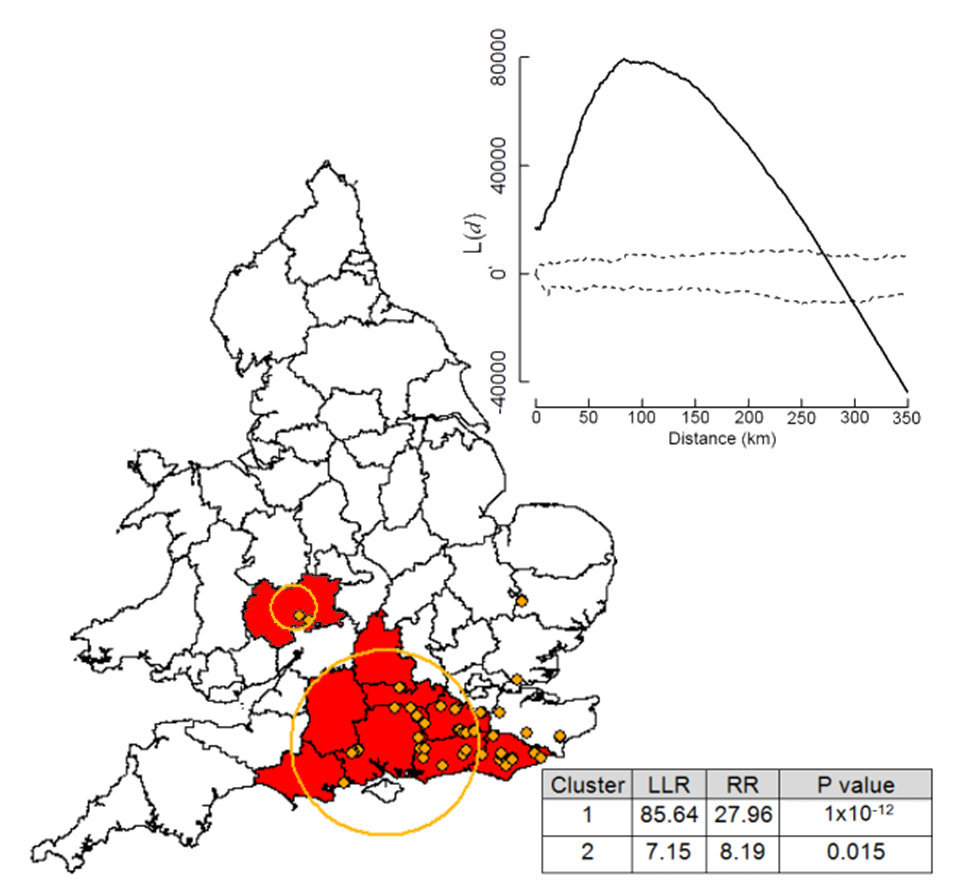** | **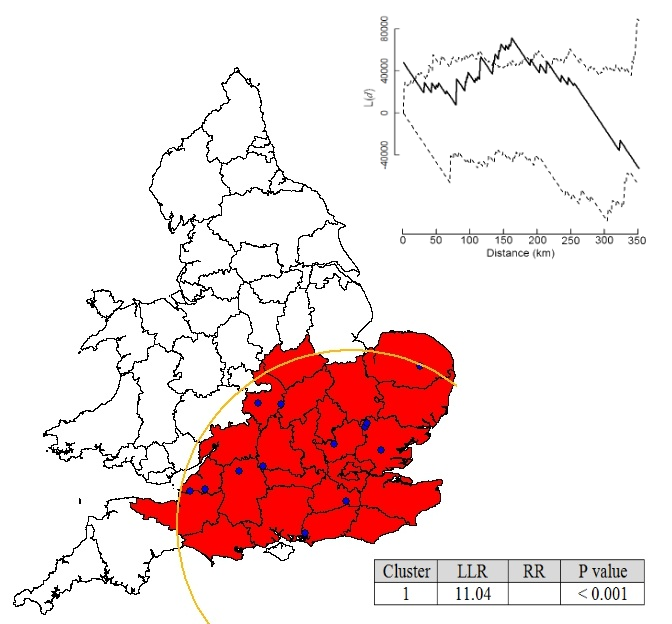** |
| **2010** | **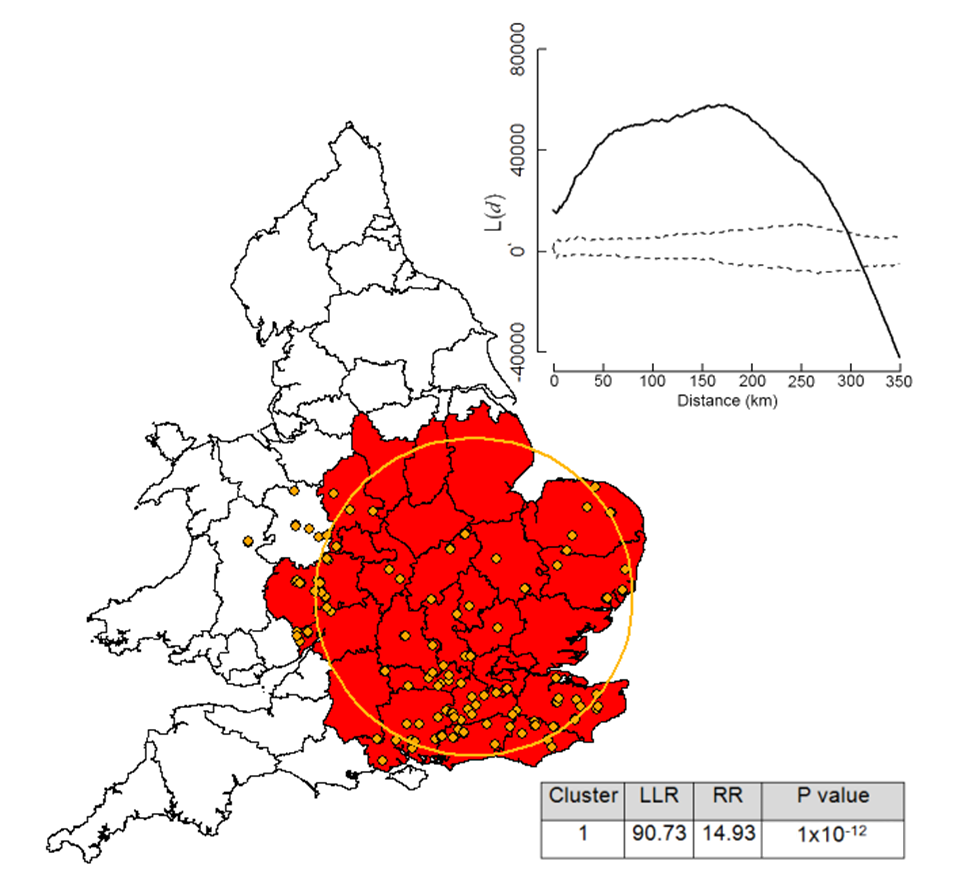** | **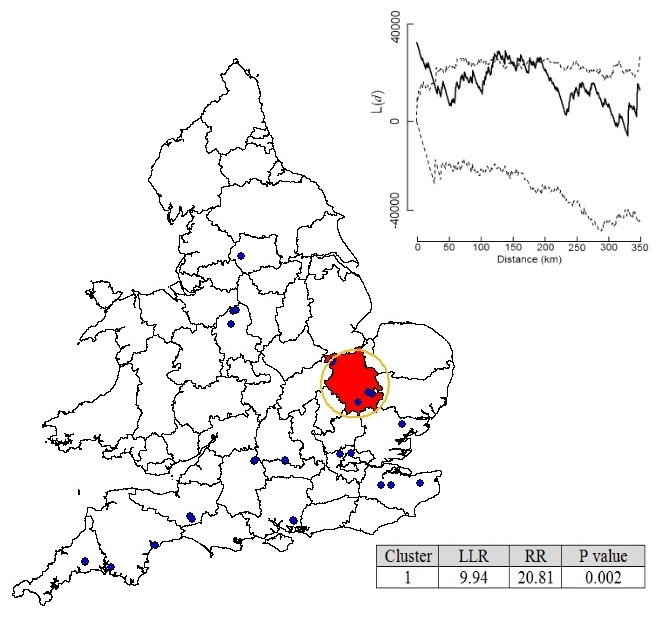** |
