# Supplementary material for: Emergence of a Novel Avian Pox Disease in British Tit Species
Source: PLoS One. 2012 Nov 21;7(11):e40176. doi: 10.1371/journal.pone.0040176 (PMC3504035; doi:10.1371/journal.pone.0040176)
Supplement: Table S1 — Great tit cases confirmed with avian pox (2006–2010). (DOC) [file pone.0040176.s005.doc]

**Supporting Information Table 1:**

**Great tit cases confirmed with avian pox at post mortem examination (2006-2010).**

| **Case** | **Month/ Year** | **County** | **Age** | **Sex^1^** | **Body condition** | **Body weight (g)** | **Diagnosis** | **Dry or wet pox** | **Description of skin lesions present (size in mm)** | **Lesion significance?**  **Other cause of death** |
| --- | --- | --- | --- | --- | --- | --- | --- | --- | --- | --- |
| 1 | Sep-06 | East Sussex | Adult | U | Thin | 19.0 | PCR | Dry | (1) Large swelling over scalp with pink/ yellow-coloured caseous core (ca. 1/3 size of head, measurements n.a.) | Single eye obscured  Predation |
| 2 | Mar-07 | Surrey | Adult | M | Thin | 16.4 | PCR  Histology | Suspect Wet | (1) Extensive ruptured scalp lesion with yellow-coloured caseous core (2) Lesion at commissure of beak on L. side (ca. 4 x 4 x 2) (3) Ruptured and haemorrhagic lesion on R. side of face (measurements n.a.) | Single eye obscured |
| 3 | Sep-07 | Kent | Adult | U | Normal | Not done | PCR  Histology | Dry | (1) Large swelling over scalp (ca. 1/3 size of head, measurements n.a.) with pink/ yellow-coloured semi-fluid core (2) Small flesh-coloured lesion on ventral aspect of R. carpus (2 x 2 x 2) | Single eye obscured |
| 4 | Oct-07 | East Sussex | Adult | U | Normal | 20.5 | PCR  Electron microscopy Histology | Dry | (1) Large pedunculated mass (16 x 13 x 10) on dorsal aspect of L. antebrachium with central yellow-coloured caseous core (2) Pedunculated mass on medial aspect of R. propatagium (9 x 5 x 3) (3) Diffuse scalp lesion with minimal swelling. | Likely interference with flight  Predation |
| 5 | Sep-07 | Surrey | Adult | U | Normal | 17.6 | PCR  Histology | Dry | (1) Large skin lesion on L. side of face (18 x 13 x 8) with ulcerated surface and haemorrhage. (2) Large skin lesion on R. side of face (10 x 7 x 9) | Single eye obscured  Euthanasia |
| 6 | Oct-07 | Worcestershire | Adult | F | Normal | 20.3 | PCR  Histology | Dry | (1) Large lesion over scalp and R. side of face with excoriated surface (19 x 18 x 22); bright yellow-coloured caseous core | Single eye obscured  Predation |
| 7 | Oct-07 | Worcestershire | Adult | M | Normal | 19.2 | PCR Electron microscopy  Histology | Dry | (1-2) Lesions on R. side of face (both 12 x 15 x 12) (3) Small lesion on nape of neck (2 x 2 x 2) | Single eye obscured  Predation |
| 8 | Oct-08 | Kent | Adult | U | Normal | 21.8 | PCR | Dry | (1) Large lesion on rostral scalp extending to L. side of face with ulcerated and haemorrhagic surface (2) Flesh-coloured pedunculated lesion on dorsal aspect of R. antebrachium (ca. 15 x 10 x 10) (3) Lesion on dorsal aspect of R. hock (ca. 5 x 5 x 5) | Single eye obscured and likely interference with flight    Predation |
| 9 | Feb-09 | Sussex | Adult | U | Thin | 20.0 | PCR | Dry | (1) Large swelling over scalp (ca. 1/3 size of head, measurements n.a.) (2) Large lesion below chin  (ca. 10 x 10 x <10) | Likely interference with feeding  *Chlamydophila* sp. infection |
| 10 | Dec-09 | Kent | Adult | F | Thin | 20.6 | PCR  Histology | Dry | (1) Large swelling over scalp (19.3 x 15.5 x18.2) with central scab and pink-coloured caseous core (2) Small lesion at posterior limit of R. eyelid (4 x 4 x 4) | Single eye obscured |
| 11 | Feb-10 | Wiltshire | Adult | F | Thin | 15.5 | PCR  Histology | Dry | (1) Large lesion on L. side of face (12.1 x 8.8 x 5) | Single eye obscured |
| 12 | Apr-10 | Surrey | Adult | F | Thin | 13.7 | PCR  Histology | Dry | (1) Pedunculated skin lesion on ventral aspect of neck (14.0 x 10.7 x <10) and (2) lesion on R. side of face, both (1-2) with surface ulceration and haemorrhage; (3) Pedunculated lesion on dorsal aspect of L. elbow with yellow-coloured caseous core (ca. 5 x 5 x 5 ) | Likely interference with flight |
| 13 | May-10 | Oxfordshire | Adult | M | Normal | 17.3 | PCR  Histology | Dry | (1) Large flesh-coloured lesion on R. side of face with surface excoriation (11.9 x 10.4 x 8.3) and (2) lesion at commissure of beak on R. side (3.8 x 2.9 x 1.6) | Single eye obscured |
| 14 | Jun-10 | Oxfordshire | Nestling (12 days old) | U | Normal | 9.5 | PCR  Histology | Dry | Multiple (<15) small yellow/ pink-coloured cutaneous nodules on the head, neck, ventral forearms and legs (ca. 3 x 2 x <2) |  |
| 15 | Oct-10 | Surrey | Adult | F | Normal | 19.0 | PCR  Histology | Dry | (1-2) Flesh coloured lesions on ventral aspect of L. carpus (largest ca. 8 x 8 x <8) and (3) on R. carpus (ca. 8 x 8 x <8); (4) Small lesion on lateral aspect of R. hock and (5) Lesion at commissure of beak on R. side (ca. 2 x 2 x 2) | Likely interference with flight  Predation |
| 16 | Oct-10 | Berkshire | Adult | U | Thin | 14.0 | PCR  Histology | Dry | (1) Extensive ruptured skin lesion along L. side of neck with yellow-coloured caseous core (measurements n.a.) | Likely restricted movement in neck  Predation |
| 17 | Oct-10 | Berkshire | Adult | F | Thin | 18.6 | PCR  Histology | Dry | (1) Lesion on head and (2) body (measurements n.a.) | Euthanasia |
| 18 | Nov-10 | Norfolk | Adult | U | Thin | 15.0 | PCR  Histology | Dry | (1) Diffuse large swelling beneath chin extending to R. commissure of beak (measurements n.a.) | Likely interference with feeding |
| 19 | Nov-10 | Warwickshire | Adult | U | Thin | 16.5 | PCR  Histology | Dry | (1) Large submandibular skin lesion (ca. 10 x 10 x 10) | Likely interference with feeding |
| 20 | Nov-10 | Cambridgeshire | Adult | U | Thin | 18.0 | PCR  Histology | Dry | (1) Extensive lesion on R. side of face extending across submandibular region with severely ulcerated and haemorrhagic surface (ca. 15 x 10 x 10) | Likely interference with feeding  Predation |

^1^ M = male; F = female; U = unknown
