# Supplementary material for: Emergence of a Novel Avian Pox Disease in British Tit Species
Source: PLoS One. 2012 Nov 21;7(11):e40176. doi: 10.1371/journal.pone.0040176 (PMC3504035; doi:10.1371/journal.pone.0040176)
Supplement: Table S2 — Samples of avian pox skin lesions from non-Paridae species. (DOC) [file pone.0040176.s006.doc]

**Supplementary Information Table 2:**

**Samples of avian pox skin lesions from non-Paridae** species

| **ZSL Case No.** | **Host** | **Date** | **County** | **Genbank Accession No.** |
| --- | --- | --- | --- | --- |
| 782-10 | House sparrow | Aug 2010 | Dorset, England | JQ067671 |
| 1004-11 | House sparrow | Oct 2011 | Shropshire, England | JQ067671 |
| 849-08 | Rock pigeon | Aug 2008 | Greater London, England | JQ067670 |
| 1583-04 | Dunnock | Sept 2004 | Leicestershire | JQ067666 |
| 935-06 | Dunnock | Aug 2006 | Warwickshire, England | JQ067666 |
| 979-09 | Dunnock | Sept 2009 | Somerset, England | JQ067666 |
| 754-11 | Dunnock | July 2011 | Hertfordshire, England | JQ067666 |
| 777-11 | Dunnock | August 2011 | Greater London, England | JQ067666 |
| 857-11 | Dunnock | August 2011 | Norfolk, England | JQ067666 |
| 870-11 | Dunnock | August 2011 | Oxfordshire, England | JQ067666 |
| 19-10 | Starling | Jan 2010 | Gwynedd, Wales | JQ067668 |
| 886-10 | Common wood-pigeon | Oct 2010 | Greater London, England | JQ067669 |
| 1139-09 | Common wood-pigeon | Nov 2009 | West Sussex, England | JQ067667 |
| B304924 | Common wood-pigeon | Feb 2006 | Fife, Scotland | JQ067667 |
| B305133 | Common wood-pigeon | May 2006 | Glasgow City, Scotland | JQ067667 |
| B307135 | Common wood-pigeon | June 2011 | Argyll & Bute | JQ067667 |
| XT850-11 | Common wood-pigeon | Aug 2011 | Herefordshire, England | JQ067667 |
