# Supplementary material for: Emergence of a Novel Avian Pox Disease in British Tit Species
Source: PLoS One. 2012 Nov 21;7(11):e40176. doi: 10.1371/journal.pone.0040176 (PMC3504035; doi:10.1371/journal.pone.0040176)
Supplement: Table S3 — Genbank entries used in the phylogeny constructed on the 4b avian pox core protein gene. (DOC) [file pone.0040176.s007.doc]

**Supporting Information Table 3:**

Genbank entries used in the phylogeny constructed on the 4b avian pox core protein gene

| **Host Family** | **Host Species** | **Origin** | **Genbank** | **Publication** |
| --- | --- | --- | --- | --- |
| Ardeidae | Great blue heron  *Ardea herodias* | USA | DQ131898 | [1] |
| Burhinidae | Stone curlew *Burhinus oedicnemus* | United Arab Emirates | AY530310 | [2] |
| Columbidae | Mourning dove  *Zenaida macroura* | USA | DQ131897 | [1] |
| Columbidae | Pigeon poxvirus strain TP-2 | Unknown | AY530303 | [2] |
| Columbidae | Rock pigeon  *Columbia livia* | UK | AM050385 | [3] |
| Columbidae | Rock pigeon  *Columbia livia* | UK | AM050386 | [3] |
| Columbidae | Wood pigeon  *Columba palumbus* | Norway | AY453177 | [4] |
| Fringillidae | Canary *Serinus canaria* | Unknown | AY530309 | [2] |
| Fringillidae | Canary *Serinus canaria* | UK | AM050375 | [3] |
| Fringillidae | House finch  *Carpodacus mexicanus* | USA | DQ131896 | [1] |
| Fringillidae | Northern cardinal  *Cardinalis cardinalis* | USA | DQ131899 | [1] |
| Falconidae | Falcon  *Falco* sp. | United Arab Emirates | AY530306 | [2] |
| Falconidae | Falcon  *Falco* sp. | United Arab Emirates | AM050376 | [3] |
| Mimidae | Mockingbird  *Mimus polyglottos* | USA | DQ131895 | [1] |
| Otididae | Houbara bustard *Chlamydotis undulata* | United Arab Emirates | AM050381 | [3] |
| Paridae | Great tit  *Parus major* | Norway | AY453173 | [4] |
| Paridae | Great tit *Parus major* | Hungary | EF634351 | [5] |
| Passeridae | House sparrow  *Passer domesticus* | Germany | AY503307 | [2] |
| Passeridae | House sparrow *Passer domesticus* | Germany | AY503308 | [2] |
| Passeridae | House sparrow  *Passer domesticus* | UK | AM050389 | [3] |
| Passeridae | House sparrow  *Passer domesticus* | UK | AM050390 | [3] |
| Phasianidae | Chicken  *Gallus gallus* | UK | AM050377 | [3] |
| Phasianidae | Turkey  *Meleagris gallopavo* | Italy | AM050388 | [3] |
| Phasianidae | Turkey  *Meleagris gallopavo* | Germany | AY530304 | [2] |
| Phasianidae | Turkey *Meleagris gallopavo* | UK | AM050387 | [3] |
| Phasianidae | FWPV vaccine | Europe | AM050380 | [3] |
| Phasianidae | FWPV vaccine | Australia | AM050378 | [3] |
| Phasianidae | FWPV vaccine | Unknown | AY530302 | [2] |
| Phasianidae | FWPV vaccine | Unknown | AY453171 | [4] |
| Phasianidae | FWPV vaccine | Europe | AM050379 | [3] |
| Polioptilidae | Blue-gray gnatcatcher  *Polioptila caerulea* | USA | DQ131900 | [1] |
| Psittacidae | Parrot  *Amazona* sp. | UK quarantine, (S. America) | AM050383 | [3] |
| Psittacidae | Love bird  *Agapornis* sp. | Germany | AY530311 | [2] |
| Struthionidae | Ostrich *Struthio camelus* | Unknown | AY530305 | [2] |
| Sturnidae | Starling  *Sturnus vulgaris* | UK | AM050391 | [3] |
| Turdidae | American robin  *Turdus migratorius* | USA | DQ131902 | [1] |
| - | Vaccinia virus (strain WR) | Unknown | M11079 | [6] |

**Supporting Information References**

[1] Adams CJ, Feldman SH, Sleeman JM (2005) Phylogenetic analysis of avian poxviruses among free-ranging birds of Virginia. Avian Dis 49 :601-605.

[2] Lüschow D, Hoffman T, Hafez HM (2004) Differentiation of avian poxvirus strains on the basis of nucleotide sequences of 4b gene fragment. Avian Dis 48: 453-462.

[3] Jarmin S, Manvell R, Gough RE, Laidlaw SM, Skinner MA (2006) Avipoxvirus phylogenetics: identification of a PCR length polymorphism that discriminates between the two major clades. J Gen Virol 87: 2191-2201.

[4] Weli SC, Traavik T, Tryland M, Coucheron DH, Nilssen O (2004b) Analysis and comparison of the 4b core protein gene of avipoxviruses from wild birds: evidence for interspecies spatial phylogenetic variation. Arch Virol 149: 2035-2046.

[5] Palade EA, Biró N, Dobos-Kovács M, Demeter Z, Mándoki M, et al. (2008) Poxvirus infection in Hungarian great tits (*Parus major*). Acta Veterinaria Hungarica 56: 539-546.

[6] Rosel J, Moss B (1985) Transcriptional and translational mapping and nucleotide sequence analysis of a vaccinia virus gene encoding the precursor of the major core polypeptide 4b. J Virol 56: 830-838.
